# Dual Inhibition of TNF-α and IL-6R mitigates cytokine release syndrome via protection of endothelial integrity and reduction of organ damage in mouse models

**DOI:** 10.3389/fimmu.2026.1848290

**Published:** 2026-07-10

**Authors:** Hyeyun Won, Eunyoung Park, Yun-Gil Roh, Mincheol Kang, Nari Kim

**Affiliations:** Prestige BioPharma IDC, Busan, Republic of Korea

**Keywords:** bispecific antibody, cytokine release syndrome, IDC007, immunotherapy-induced toxicity, interleukin-6 receptor, Tumor Necrosis Factor-alpha

## Abstract

**Introduction:**

Cytokine Release Syndrome (CRS) is a life-threatening complication of T-cell-engaging immunotherapies, causing vascular leakage and multi-organ dysfunction. Standard Interleukin-6 (IL-6) blockade often fails in severe cases by leaving the upstream initiator, Tumor Necrosis Factor-α (TNF-α), unchecked. TNF-α drives early macrophage activation and endothelial injury, whereas IL-6 amplifies systemic inflammation. To decisively interrupt this inflammatory feedback loop, we developed IDC007, a novel bispecific antibody simultaneously neutralizing TNF-α and IL-6 receptor (IL-6R).

**Methods:**

We evaluated the neutralizing effects of IDC007 in an in vitro CRS model utilizing OKT3/R848-stimulated human peripheral blood mononuclear cells and human umbilical vein endothelial cells. Furthermore, we utilized an OKT3-induced humanized CRS mouse model to assess in vivo target engagement and therapeutic efficacy.

**Results:**

In vitro, IDC007 effectively suppressed pro-inflammatory cytokine secretion (e.g., TNF-α, IFN-γ) and prevented endothelial barrier dysfunction. In vivo, IDC007 successfully engaged both targets. Administration mitigated physiological deterioration, including hypothermia and weight loss, leading to improved survival. Dual inhibition effectively prevented immune hyperactivation, evidenced by the significant attenuation of splenomegaly and lung-specific organ damage.

**Discussion:**

These findings serve as a preclinical proof-of-concept demonstrating that dual targeting of TNF-α and IL-6R offers a potent mechanistic approach to attenuate severe CRS by preserving vascular endothelial integrity and overcoming the therapeutic limitations of conventional monotherapies.

## 1 Introduction

Cytokine Release Syndrome (CRS) is a systemic inflammatory response that frequently arises as a life-threatening adverse event associated with T-cell-engaging immunotherapies, including chimeric antigen receptor (CAR) T-cell therapies and bispecific T-cell engagers (BiTEs) (1, 2). While these modalities have revolutionized cancer treatment by harnessing the potency of the host immune system, the rapid and massive release of pro-inflammatory cytokines—triggered by the synergistic activation of T cells and bystander myeloid cells—can lead to severe clinical manifestations. These range from high-grade fever and hypotension to capillary leak syndrome, multi-organ dysfunction, and potential lethality (3, 4). The pathophysiology of severe CRS is characterized by a “cytokine storm” a complex network of feed-forward signaling loops involving key mediators such as interleukin-6 (IL-6), tumor necrosis factor-alpha (TNF-α), and interferon-gamma (IFN-γ) (5).

Currently, the blockade of IL-6 signaling, utilizing agents like tocilizumab (an anti-IL-6 receptor antibody), serves as the standard of care for managing CRS (6). However, clinical evidence suggests that IL-6 blockade alone may be insufficient for all patients, particularly those with severe, rapid-onset CRS or those exhibiting resistance to tocilizumab (7). This limitation stems largely from the fact that IL-6 acts as a downstream effector, whereas upstream cytokines, specifically TNF-α, initiate the inflammatory cascade and independently contribute to endothelial activation and vascular permeability (8, 9). Recent preclinical studies have highlighted that macrophage-derived TNF-α plays a critical role in the early phase of CRS, suggesting that targeting this upstream initiator may prevent the exponential amplification of the cytokine storm (10). Therefore, a therapeutic strategy that simultaneously neutralizes the upstream initiator (TNF-α) and the downstream effector (IL-6) holds the promise of interrupting the inflammatory amplification loop more effectively than monotherapy.

In this study, we describe the development and characterization of IDC007, a novel IgG-based appended bispecific antibody designed to simultaneously target TNF-α and IL-6R. We chose tocilizumab and adalimumab as the parent antibodies due to their strong clinical relevance; tocilizumab is the current FDA-approved standard of care for severe CRS, while adalimumab is a highly potent anti-TNF-α agent often utilized off-label for severe, refractory inflammation. By combining these clinical assets, we hypothesized that bridging these two targets would not only provide superior control of the cytokine storm but also offer distinct advantages in preserving vascular integrity and reducing off-target toxicity compared to conventional combination therapies. To rigorously evaluate the efficacy of IDC007, we established a robust *in vitro* CRS model utilizing human peripheral blood mononuclear cells (PBMCs) stimulated with an anti-CD3 antibody (OKT3) and the Toll-like receptor (TLR) 7/8 agonist R848. This dual-stimulation approach was specifically employed to mimic the severe macrophage activation and dysregulated innate immune responses observed in high-grade clinical CRS, overcoming the limitations of T-cell stimulation alone (10, 11).

Here, we demonstrate that IDC007 effectively suppresses the secretion of multiple pro-inflammatory cytokines and prevents endothelial barrier dysfunction, a key driver of organ damage, by preserving VE-cadherin integrity. Furthermore, simultaneous targeting of both proteins in CRS mouse models resulted in reduced organ pathology and a faster restoration of physiological parameters, including body temperature and weight. Overall, this study serves as a preclinical proof-of-concept validating the mechanistic rationale of simultaneous TNF-α and IL-6R blockade for immunotherapy-induced CRS.

## 2 Materials and methods

### 2.1 Cell culture

Human umbilical vein endothelial cells (HUVECs) (Lonza, C2519A) were maintained in endothelial basal medium-2 (EBM-2, Lonza, CC-3156) supplemented with the EGM-2 SingleQuot Kit (Lonza, CC-4176) according to the manufacturer’s instructions. This included 2% (v/v) FBS, human fibroblast growth factor-B (hFGF-B), vascular endothelial growth factor (VEGF), R3-insulin-like growth factor-1 (R3-IGF-1), ascorbic acid, human epidermal growth factor (hEGF), gentamicin/amphotericin B (GA-1000), heparin, and hydrocortisone. Cultures were incubated at 37 °C in a humidified atmosphere containing 5% CO_2_, passaged at 80–90% confluency using a 1:3 split ratio with TrypLE (Gibco, 12604013), and utilized between passages 4 and 8 to preserve endothelial phenotype.

Primary human PBMCs were obtained from cryopreserved leukopaks (Human Peripheral Blood Leukopak, Frozen; STEMCELL Technologies, 200-0131). Leukopaks were thawed according to the manufacturer’s protocol using rapid thawing in a 37 °C water bath, followed by dilution with pre-warmed thawing medium containing DNase I (STEMCELL, 7470). Thawed PBMCs were washed twice (300 × g, 10 min, room temperature) and aliquoted at 2 × 10^7^ cells/vial in freezing medium consisting of 90% (v/v) Fetal Bovine Serum (FBS; Cytiva, SH30919.03) and 10% (v/v) Dimethyl sulfoxide (DMSO; Sigma, D2438) for storage in liquid nitrogen. Complete RPMI 1640 medium consisted of RPMI 1640 (Gibco, 11875-093) supplemented with 10% (v/v) FBS and 1× antibiotic-antimycotic (Gibco, 15240062). Cells were cultured in complete RPMI 1640 at 37 °C and 5% CO_2_, with post-thaw viability exceeding 90% as assessed by trypan blue exclusion.

THP-1 monocytes (KCLB, 40202) were maintained in RPMI 1640 supplemented with 300 mg/L L-glutamine (Gibco, 25030081), 25 mM HEPES (Gibco, 15630-080), 10% (v/v) FBS, 1× antibiotic-antimycotic (Gibco, 15240062), and 50 μM β-mercaptoethanol (Sigma, 63689). Cells were passaged every 3–4 days by dilution to maintain a density of 2–8 × 10^5^ cells/mL (1:5 split ratio), with viability consistently exceeding 95% as determined by trypan blue exclusion.

WEHI-13VAR cells (ATCC, CRL-2148) were maintained in RPMI 1640 medium supplemented with 10% (v/v) heat-inactivated FBS, 2 mM L-glutamine (Gibco, 25030081), 1× antibiotic-antimycotic (Gibco, 15240062), and 50 μM β-mercaptoethanol (Sigma, M6250). Cells were cultured at 37 °C in a humidified atmosphere containing 5% CO_2_, passaged every 3–4 days by dilution (1:5 split ratio) to maintain density between 2-8 × 10^5^ cells/mL, with viability consistently exceeding 90% as determined by trypan blue exclusion.

### 2.2 Binding affinity analysis by SPR

Biacore 8K surface plasmon resonance (Cytiva, 29722782) analysis was performed to determine the binding kinetics and affinity (K_D_) of IDC007, Adalimumab, and Tocilizumab to their respective targets, human TNF-α and IL-6R. A Series S Sensor Chip Protein A (Cytiva, 29127555) was activated at room temperature for 24 hours prior to docking into the instrument. The running buffer was prepared by diluting 10× HBS-EP+ buffer (Cytiva, BR100826) to a 1× concentration and filtering. For the assay, the antibodies (ligands) were diluted to 10 nM in the running buffer and captured onto the Protein A chip surface. Recombinant human TNF-α and IL-6R proteins (analytes) were prepared at an initial concentration of 50 nM and serially diluted two-fold to generate a concentration range of 3.125 to 50 nM (0, 3.125, 6.25, 12.5, 25, and 50 nM). The binding interactions were measured using a multi-cycle kinetics approach. The association (K_on_) and dissociation (K_off_) rate constants were derived from the sensorgrams, and the equilibrium dissociation constant (K_D_) was calculated using the dedicated Biacore™ insight evaluation software (Cytiva, 3.0.12.15655 version).

### 2.3 Binding affinity analysis by Enzyme-linked immunosorbent assay (ELISA)

#### 2.3.1 TNF-α ELISA

96-well plates were coated with TNF-α (Gibco, PHC3011) at 250 ng/mL in carbonate-bicarbonate buffer (100 μL/well) overnight at 4 °C. Plates were washed three times with 1X TBST (Thermo Scientific, 28360; 350 μL/well), blocked with SuperBlock™ (ScyTek Laboratories, AAA99; 200 μL/well) for 1 h at room temperature (RT), and incubated with test antibodies (serial 1:5 dilutions from 50, 000 pM to 0.128 pM in TBST; 100 μL/well) for 2 h at RT. Following three washes with 1X TBST, plates were incubated with goat anti-human IgG Fc-HRP (Invitrogen, 31413; 80 ng/mL) or goat anti-human kappa light chain-HRP (Invitrogen, A18853; 500 ng/mL) (100 μL/well) for 1 h at RT. After three additional washes, plates were developed with TMB substrate (Thermo Scientific, 34029; 100 μL/well) for 5 min at RT, stopped with stop solution (Surmodics, LSTP-1000-01; 100 μL/well), and absorbance was measured at 450 nm.

#### 2.3.2 IL-6R ELISA

96-well plates were coated with rhIL-6Rα (Peprotech, 200-06RC) at 1 μg/ml in carbonate-bicarbonate buffer (100 μL/well) overnight at 4 °C. Plates were washed 3× with TBST (350 μL/well), blocked with SuperBlock™ (200 μL/well) for 1 h at RT, and incubated with test antibodies (serial 1:5 dilutions from 725, 000 pM to 0.371 pM in TBST; 100 μL/well) for 2 h at RT. After washing 3× with TBST, plates were incubated with goat anti-human IgG Fc-HRP (80 ng/mL) or goat anti-human kappa light chain-HRP (800 ng/mL) (100 μL/well) for 1 h at RT. Plates were washed 3× with TBST, developed with TMB substrate (100 μL/well) for 5 min at RT, stopped with stop solution (100 μL/well), and absorbance was measured at 450 nm. EC_50_ values were calculated from absorbance versus antibody concentration curves using four-parameter logistic regression in GraphPad Prism (v10.0) for both assays.

### 2.4 TNF-α neutralization assay

TNF-α neutralization activity was assessed using Actinomycin D-sensitized WEHI-13VAR cells in assay medium consisting of RPMI 1640 supplemented with 2% (v/v) FBS. Serially diluted antibodies (final concentrations 6.836–3500 pM; 100 μL/well) were added to 96-well plates, followed by human TNF-α (NIBSC, 12/154; final 24.84 IU/ml, 50 μL/well). Cells (5 × 10^4^/well in assay medium containing 2 μg/ml Actinomycin D, Sigma, A9415) were seeded in triplicate and incubated for 20 h at 37 °C, 5% CO_2_. CellTiter 96^®^ AQueous One Solution MTS (Promega, G5430; 20 μL/well) was added, plates incubated for an additional 5 h, and absorbance measured at 490 nm. EC_50_ values were calculated from dose-response curves using four-parameter logistic regression in GraphPad Prism.

### 2.5 IL-6R neutralizing antibody assay

IL-6R neutralizing activity was assessed using IL-6 Bioassay Cells (Promega, J3025). Frozen cells were rapidly thawed in a 37 °C water bath for 2 min and seeded at 2.5 × 10^4^ cells/well (50 μL/well in Bioassay Medium: RPMI 1640 + 10% FBS) into white, flat-bottom 96-well assay plates (Corning, 3917). Test antibodies (final concentrations 0.02–1500 nM) were added to seeded cells and pre-incubated for 20 min at 37 °C, 5% CO_2_. Recombinant human IL-6 (PeproTech, 200-06; final 120 ng/mL) was then added, and cells were incubated for an additional 6 h at 37 °C, 5% CO_2_. Bio-Glo™ Luciferase Assay Reagent (Promega, G7940; 75 μL/well) was added, plates incubated for 10 min at room temperature, and luminescence measured using a plate luminometer (1 s integration/well). IC_50_ values were calculated from dose-response curves using four-parameter logistic regression in GraphPad Prism.

### 2.6 *In vitro* CRS induction

OKT3 (BioXCell, BE0001-2) was diluted to 1 μg/mL in PBS and coated onto 24-well plates (1 mL/well) by incubation at 37 °C for 1 h. Plates were washed twice with PBS. Human PBMCs were seeded at 2 × 10^6^ cells/well in RPMI 1640 (Gibco, 11875-093) supplemented with 10% (v/v) FBS and 1× antibiotic-antimycotic. R848 was added at final concentrations of 15.6, 62.5, or 250 ng/mL to achieve a total volume of 1 mL/well. Following 24 h incubation at 37 °C, 5% CO_2_, conditioned medium (CM) was collected for ELISA analysis. R848 at 250 ng/mL was determined optimal for CRS induction and used thereafter.

For antibody treatment experiments, IDC007 was produced in-house, Tocilizumab (Selleckchem, A2012) was purchased from Selleckchem, and adalimumab biosimilar (PBP1502) was provided by Prestige Biopharma. OKT3-coated wells were treated with R848 (final 250 ng/mL), antibodies (final 500 nM), and PBMC suspension (2 × 10^6^ cells/well) to a final volume of 1 mL/well. Following 24 h incubation at 37 °C, 5% CO_2_, conditioned medium was collected, and CRS induction was confirmed by ELISA prior to use in downstream assays.

### 2.7 Cytokine quantification by ELISA

Concentrations of TNF-α, IL-6, and IFN-γ in cell culture supernatants were quantified using commercial ELISA kits: Human TNF-α DuoSet ELISA (R&D Systems, DY210), Human IL-6 DuoSet ELISA (R&D Systems, DY206), and Human IFN-γ DuoSet ELISA (R&D Systems, DY285B), according to the manufacturers’ protocols. IL-6R concentrations were measured by sandwich ELISA using the Human IL-6R alpha DuoSet ELISA kit (R&D Systems, DY227) with tocilizumab (Selleckchem, A2012) substituted for the capture antibody, following the manufacturer’s protocol. Standards and samples were diluted in 1× reagent diluent 2 (R&D Systems, DY995). Due to the use of tocilizumab as the capture antibody, this assay specifically quantifies unbound free sIL-6R, as therapeutic antibodies (IDC007 or tocilizumab) present in the samples competitively mask the binding epitope. Optical density was measured at 450 nm with wavelength correction at 540 nm, and concentrations were calculated from four-parameter logistic standard curves.

### 2.8 Immunocytochemistry

Glass coverslips (Marienfeld Superior, 111520) were sterilized by immersion in 100% ethanol, flamed using an alcohol lamp, and placed in 24-well plates. Coverslips were coated with rat tail collagen I (Gibco, A1048301; 7.5 μg/cm²) by incubation at 37 °C for 1 h, followed by air-drying for 30 min and washing with Dulbecco’s phosphate-buffered saline (DPBS) (Welgene, LB 011-02). HUVECs were seeded at 1 × 10^5^ cells/well on coated coverslips and cultured for 48 h to form monolayers. Cells were treated with 2% (v/v) conditioned medium from OKT3/R848-stimulated hPBMCs with or without antibodies for 24 h. Cells were fixed with 4% paraformaldehyde (Biosolution, BP031) for 20 min at RT, permeabilized with 0.1% Triton X-100 (Sigma, 93443) in PBS for 10 min, and blocked with 5% BSA (Sigma, A1595) in 0.1% Triton X-100/PBS for 30 min at RT. VE-cadherin was stained with Alexa Fluor^®^ 488 anti-human VE-cadherin/CD144 (Invitrogen, 53-1449-42), followed by nuclear counterstaining with 4′, 6-diamidino-2-phenylindole (DAPI) (Sigma, D9542; 1:200 dilution) for 10 min at RT. After DPBS washes, coverslips were mounted onto glass slides using ProLong Gold Antifade Mountant (Invitrogen, P36930). Images were acquired using a ZEISS LSM 900 confocal laser scanning microscope, and VE-cadherin junctional thickness at cell-cell contacts was quantified from fluorescence images using ImageJ software.

### 2.9 HUVEC vascular permeability assay

Endothelial permeability was assessed using collagen I–coated 6.5 mm Transwell inserts with 3.0 μm pore polycarbonate membranes (Costar, 3415). HUVECs (1 × 10^5^ cells/insert) were cultured for 48 h to form confluent monolayers and then treated for 24 h with 2% (v/v) conditioned medium from OKT3/R848-stimulated hPBMCs with or without antibodies. FITC–dextran (10 μg/mL, 150 μL) was added to the upper chamber, with 600 μL assay medium in the lower chamber. After incubation, fluorescence in the lower chamber was measured (Victor Nivo 3, Ex/Em 480/30–530/30 nm), and permeability was expressed as FITC–dextran fluorescence relative to the non-CRS control.

### 2.10 Monocyte adhesion assay

Wells were coated with rat tail collagen I (7.5 μg/cm²), incubated at 37 °C for 1 h. HUVECs were seeded at 1 × 10^4^ cells/well in collagen I–coated 96-well plates and cultured for 48 h at 37 °C to form confluent monolayers. To generate CRS-mimicking conditions, HUVEC monolayers were treated with 2% (v/v) conditioned medium from OKT3/R848-stimulated hPBMCs with or without antibodies for 24 h. After treatment, the medium was replaced with serum-free EBM-2, and cells were incubated for an additional 4 h at 37 °C. THP-1 monocytes were labeled with CellTrace™ CFSE (Cell Proliferation Kit, Invitrogen, C34554) according to the manufacturer’s instructions and added to HUVEC monolayers at 2 × 10^5^ cells/well. To accurately quantify cell adhesion as the final readout, the co-cultures were incubated for 3 h at 37 °C. Subsequently, non-adherent THP-1 cells were removed by gently washing the wells three times with EBM-2, ensuring the adherent monolayer remained undisturbed. The final quantitative readout was obtained by measuring the fluorescence of the stably adherent CFSE-labeled THP-1 cells using a Victor Nivo 3 plate reader (PerkinElmer; excitation 480/30 nm, emission 530/30 nm). The degree of monocyte adhesion was then expressed as relative fluorescence intensity normalized to the CRS-treated IgG control.

### 2.11 *In Vivo* CRS models and treatment regimens

Six-week-old female C57BL/6J and NOD.Cg-Prkdc^scid^ Il2rg^tm1Wjl^/SzJ (NSG) mice were purchased and acclimatized for one week prior to experimentation. Mice were used at 7 weeks of age, with five mice randomly assigned to each experimental group (n=5). To establish a syngeneic CRS model, 7-week-old female C57BL/6J mice were intravenously (i.v.) injected with 2 mg/kg of anti-mouse CD3ϵ antibody (clone 145-2C11, BioXCell, BP0001-1) (12). To evaluate therapeutic efficacy, mice were treated with 30 mg/kg of anti-mouse TNF-α antibody (mTNF-α, BioXCell, BE0058), anti-mouse IL-6R antibody (mIL-6R, BioXCell, BE0047), or a combination of both. Treatments were administered intravenously either simultaneously with CRS induction or 2 hours post-induction. For the humanized CRS model, 7-week-old female NSG mice were engrafted with hPBMCs (2 × 10^7^ cells/mouse) via intravenous injection. One-week post-engraftment, CRS was induced by intravenous administration of the anti-human CD3 antibody (OKT3, BioXCell, BE0001) (13). A Control group received an equivalent dose of the OKT3 isotype control (BioXCell, BE0085). For therapeutic intervention, mice were intravenously administered 2.5 mg/kg of IDC007, Adalimumab, Tocilizumab, or a human IgG1 isotype control (BioXCell, BP0297). Physiological parameters, including body weight and surface temperature, were intensively monitored for 96 hours post-induction to cover the acute mortality phase. Body temperature and body weight were monitored at scheduled time points to assess physiological deterioration associated with CRS. Rectal temperature was measured using a rodent thermometer (BIOSEB LAB, BIO-TK8851). Blood samples were collected via retro-orbital sinus puncture for serum separation and subsequent cytokine analysis. For survival analysis, mice exhibiting a body weight loss of >20% compared to baseline were classified as non-survivors. These events were recorded as mortality to generate survival curves. All animal experiments were conducted in accordance with the guidelines approved by the Institutional Animal Care and Use Committee (IACUC, Protocol No. IDC-IACUC-2517-R2). The use of hPBMCs was approved by the Institutional Review Board (IRB, Protocol No. 2022-08-001).

### 2.12 Histological analysis

All surviving mice were subsequently euthanized on day 7, and their major organs (e.g., spleen, lungs) were harvested for gross evaluation and histological analysis. Lung tissues were harvested and fixed in 4% paraformaldehyde (PFA). The fixed tissues were processed using an automated Tissue Processor (Leica, 14049350667) and embedded in paraffin blocks using a Heated Paraffin Embedding Station (Leica, 14039357257) and a Cold Plate (Leica, 14039357262). Paraffin-embedded tissues were sectioned at a thickness of 5 μm using an Autocut Microtome (Leica, 149AUTO00C1). The sections were floated in a Water Bath (Leica, 14041521466) and placed onto BOND Plus Slides (Leica, S21.2113.A). After drying on a Heating Plate (Leica, 14042321474), Hematoxylin and eosin (H&E) staining was performed using an Autostainer XL Workstation (Leica Biosystems). Specifically, the sections were stained with Hematoxylin solution according to Delafield (Sigma-Aldrich, 03971) and Eosin Y solution, Alcoholic (Sigma-Aldrich, HT110132). The stained slides were cover-slipped with Matsunami cover glass (24x50 mm, No. 1, thickness 0.12–0.17 mm; HMA-C24501) and digitally imaged using an Aperio GT 450 pathology slide scanner (Leica Biosystems).

### 2.13 Quantification and pathological assessment

To evaluate the severity of lung inflammation, digital images obtained from the scanner were quantitatively analyzed using ImageJ software. To quantify the extent of inflammatory cell infiltration, the total number of cells per field of view (FOV) was counted within the inflamed areas of the lung tissue using ImageJ software. Multiple representative FOVs were randomly selected for each tissue section to calculate the average cellular density, allowing for a robust comparison of infiltration severity across the experimental groups. For the assessment of splenomegaly, the absolute weight and longitudinal length of each spleen were measured immediately after excision to determine the extent of immune activation.

### 2.14 Flow cytometry analysis

For *in vitro* experiments assessing HUVEC endothelial activation, endothelial activation markers on HUVECs were analyzed by flow cytometry. Cells were detached from monolayers using TrypLE following stimulation and treatment, washed twice with Fluorescence-activated cell sorting (FACS) buffer (2% FBS in DPBS; 400 × g, 5 min), and stained for 30 min at 4 °C in the dark with Pacific Blue anti-human intercellular adhesion molecule-1 (ICAM-1)/CD54 (BioLegend, 353110), Alexa Fluor^®^ 488 anti-human VE-cadherin/CD144 (Invitrogen, 53-1449-42), APC anti-human CD62E (BD, 551144), and corresponding isotype controls [Pacific Blue mouse IgG1, κ (BioLegend, 400151), Alexa Fluor^®^ 488 mouse IgG1, κ (Invitrogen, 53-4714-80), APC mouse IgG1, κ (BD, 555751)]. After two washings, cells were resuspended in 100 μl FACS buffer. Data acquisition was performed on a flow cytometer (Agilent, 2010064AA). Live singlets were gated (FSC/SSC, FSC-H/FSC-A), compensated, and analyzed using instrument software.

### 2.15 Serum collection and cytokine analysis

To evaluate the therapeutic efficacy of IDC007, blood samples were collected from hPBMC-engrafted mice following CRS induction and treatment administration. Blood was obtained via orbital sinus sampling using heparinized capillary tubes (Kimble Chase, HCH-41B250) and collected into serum-separating tubes (BD Biosciences, 365967). Serum was isolated by centrifugation according to the manufacturer’s instructions and stored at negative 80 °C until analysis. The concentrations of human IFN-γ, IL-6, and TNF-α in the serum were quantified using the V-PLEX Custom Human Biomarkers kit (Meso Scale Discovery, K151A9H-1). Electrochemiluminescence signals were detected and analyzed using an MSD Luminescence Spectrometer (Meso Scale Discovery, AI1AA-0). Additionally, sIL-6R levels were measured in serum samples pooled by groups using the Human IL-6R alpha DuoSet ELISA kit (R&D Systems, DY227). All assays were performed in accordance with the respective manufacturer’s protocols, and cytokine concentrations were calculated based on standard curves generated using recombinant protein standards. Due to the use of tocilizumab as the capture antibody, this assay specifically quantifies unbound ‘free’ sIL-6R, as therapeutic antibodies (IDC007 or tocilizumab) present in the samples competitively mask the binding epitope.

### 2.16 Statistical analysis and graphing

All statistical analyses were performed, and graphical representations were generated using GraphPad Prism version 10.0. For most parameters, data were presented as the mean ± SD. Unless otherwise stated, *in vitro* data represent at least three independent biological replicates (n ≥ 3), and *in vivo* data represent five mice per group (n = 5 biological replicates); *p < 0.05, **p < 0.01, ***p < 0.001, ****p < 0.0001. In *in vitro* experiments, EC_50_ and IC_50_ values were calculated from dose-response curves using four-parameter logistic (4PL) regression. Cytokine concentrations were determined from 4PL standard curves. Permeability, adhesion, and hepatotoxicity data were analyzed by one-way analysis of variance (ANOVA) with Tukey’s multiple comparisons test. In *in vivo* analyses, spleen weight and longitudinal length were specifically represented as box-and-whisker plots (min to max) to clearly illustrate the range and distribution of the splenomegaly observed across the experimental groups. Statistical significance among experimental groups was determined using a one-way ANOVA, followed by Dunnett’s multiple comparisons test. For most parameters, the CRS group served as the reference for statistical comparisons to evaluate the therapeutic efficacy of the treatments. In contrast, for the analysis of immune cell populations, comparisons were performed relative to the Control group to assess deviations from the physiological baseline.

## 3 Results

### 3.1 Generation and characterization of the bispecific antibody IDC007 Targeting TNF-α and IL-6R

**Figure 1:**
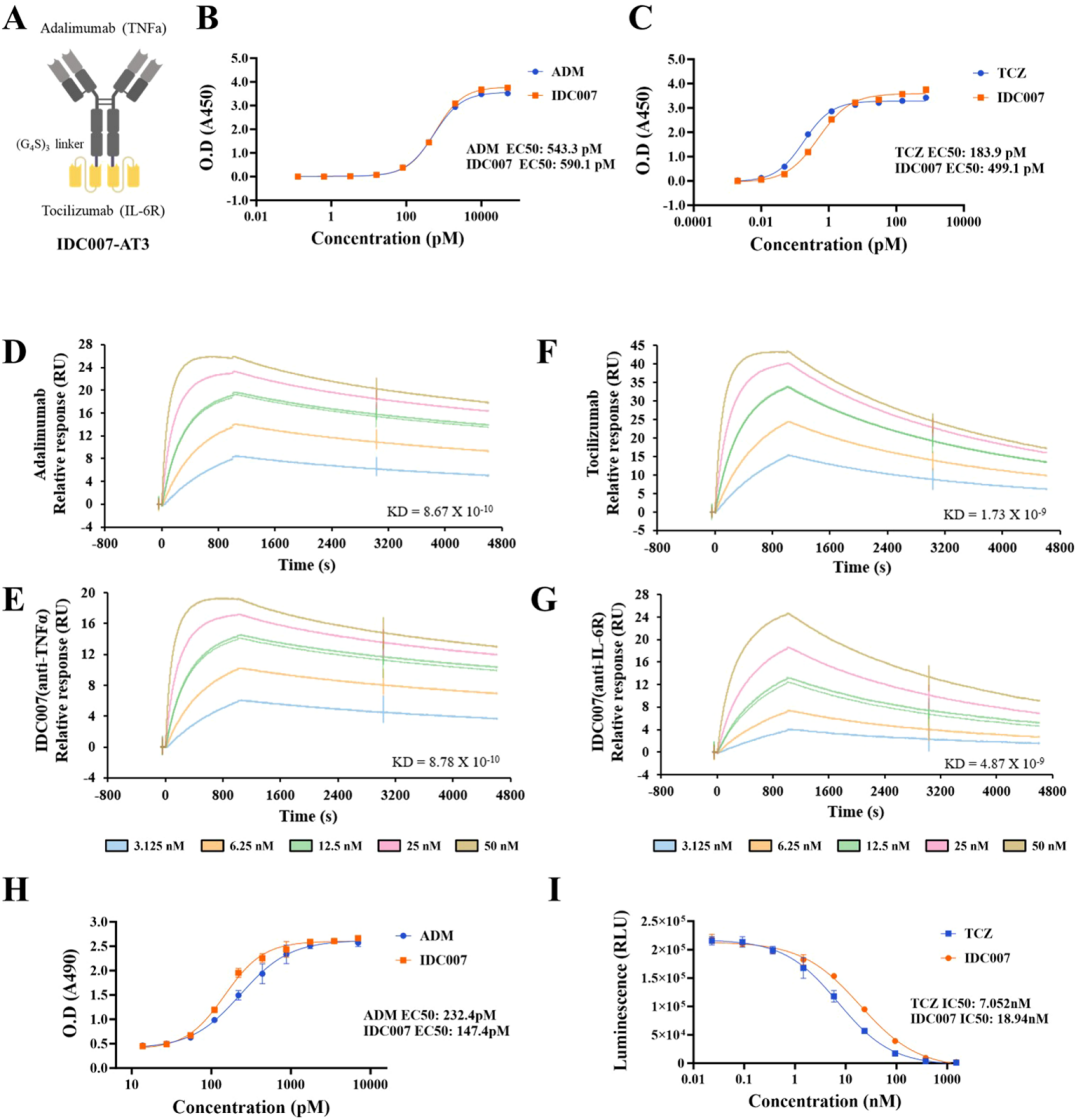
Generation and characterization of the bispecific antibody IDC007 targeting TNF-α and IL-6R. **(A)** Schematic representation of IDC007, a bispecific antibody generated by fusing the scFv of the anti–IL-6R antibody tocilizumab to the C-terminus of the heavy chain of the anti-TNF-α antibody adalimumab via a (G4S)_3_ linker. **(B, C)** ELISA-based binding analysis showing that IDC007 binds human TNF-α with an affinity comparable to adalimumab and recognizes IL-6R with slightly reduced affinity compared with the bivalent parental antibody tocilizumab. **(D–G)** Surface plasmon resonance (SPR) measurements confirming that IDC007 retains TNF-α binding kinetics similar to adalimumab, while displaying moderately lower affinity for IL-6R than tocilizumab, consistent with its scFv fusion format. **(H, I)** Cell-based functional assays demonstrating the neutralizing activity of IDC007. A human IgG isotype was included as a non-neutralizing baseline control. IDC007 more potently neutralizes TNF-α-induced cytotoxicity than adalimumab **(H)** and effectively inhibits IL-6R signaling at nanomolar concentrations **(I)**, validating IDC007 as a dual-targeting agent against TNF-α and IL-6R. Data are presented as the mean ± SD of 3 independent biological replicates (n = 3).

To simultaneously block the upstream initiator TNF-α and the downstream effector IL-6 of the cytokine storm, we generated a bispecific antibody, designated IDC007. IDC007 was constructed by fusing the single-chain variable fragment (scFv) of the anti-IL-6R antibody (tocilizumab) to the C-terminus of the heavy chain of the anti-TNF-α antibody (adalimumab) via a (G4S)3 linker (**Figure 1A**). Following the expression and purification of IDC007, we evaluated its biochemical properties and structural integrity. Sodium dodecyl sulfate-polyacrylamide gel electrophoresis (SDS-PAGE) analysis confirmed the proper assembly of the bispecific antibody, revealing a single intact band at approximately 198.8 kDa under non-reducing conditions. Under reducing conditions, the antibody dissociated into two distinct bands corresponding to the heavy chain-scFv fusion (76 kDa) and the light chain (23.4 kDa), as expected (**Supplementary Figure 1A**). Furthermore, size-exclusion fast protein liquid chromatography (SE-FPLC) demonstrated that the purified IDC007 maintained a high monomeric purity of >95.0% (**Supplementary Figure 1B**).

First, we evaluated the binding activities of IDC007 to its target antigens using ELISA. IDC007 exhibited a dose-dependent binding affinity to human TNF-α with an EC_50_ value of 590.1 pM, which was comparable to that of the parental antibody adalimumab (543.3 pM) (**Figure 1B**). For IL-6R, IDC007 showed an EC_50_ of 499.1 pM. Although this binding capacity was slightly lower than that of the bivalent parental antibody tocilizumab (183.9 pM)—a difference attributable to the structural format of the scFv moiety fused to the IgG backbone—IDC007 retained high affinity at the nanomolar level (**Figure 1C**). This confirms that the antibody possesses sufficient binding ability to specifically recognize the antigen and effectively block IL-6 signaling, demonstrating no functional compromise (**Figure 1I**).

To further quantify the binding kinetics, surface plasmon resonance (SPR) analysis was performed using a Protein A sensor chip. Antibodies (ligands) were captured at a concentration of 10 nM, followed by the injection of antigens (analytes) at concentrations ranging from 3.125 to 50 nM to monitor association and dissociation phases. The equilibrium dissociation constant (KD) of IDC007 for TNF-α was 0.878 nM, which was nearly identical to that of adalimumab (0.867 nM) (**Figures 1D, E**). For IL-6R, the K_D_ value of IDC007 was 4.87 nM, whereas tocilizumab showed a K_D_ of 1.73 nM (**Figures 1F, G**). Despite the structural difference of the IL-6R binding domain formatted as an scFv, IDC007 maintains a binding affinity in the nanomolar range, demonstrating its capability for effective and specific antigen engagement.

To rigorously validate our cell-based functional assays and establish a baseline for specificity, we first evaluated the neutralizing potency of our in-house parental antibodies alongside commercial reference drugs. As shown in **Supplementary Figure 3**, the in-house antibodies demonstrated equivalent neutralizing capacities to commercial adalimumab against TNF-α-induced cytotoxicity (EC_50_: 3.925 µg/mL vs. 3.435 µg/mL) (**Supplementary Figure 3A**) and commercial tocilizumab against IL-6-induced signaling (IC_50_: 0.2186 µg/mL vs. 0.2199 µg/mL) (**Supplementary Figure 3B**). Notably, a human IgG isotype was exclusively included in these initial comparative assays as a baseline negative control, confirming no non-specific inhibition. Having established this strict functional baseline, our subsequent evaluations directly compared the potency of the bispecific IDC007 against these validated parental antibodies. We next assessed the biological neutralizing activity of IDC007 using cell-based assays employing two distinct cell lines. In the cell-based TNF-α neutralization assay, IDC007 effectively inhibited TNF-α-induced cytotoxicity with an EC_50_ of 147.4 pM, demonstrating superior potency compared to adalimumab (232.4 pM), while the IgG control showed no protective effect against TNF-α (**Figure 1H**). Conversely, to evaluate IL-6R blockade, we utilized a luciferase reporter cell line responsive to IL-6 stimulation. In this assay, IDC007 inhibited IL-6 signaling with an IC_50_ of 18.94 nM, whereas the IgG-treated baseline group exhibited unhindered maximum luminescence (**Figure 1I**). While this potency was lower than that of tocilizumab (7.052 nM), these results confirm that IDC007 functions as a dual-targeting agent capable of neutralizing both TNF-α and IL-6R signaling pathways at nanomolar concentrations.

### 3.2 IDC007 attenuates pro-inflammatory cytokine surges in an *In Vitro* CRS model mimicking severe macrophage activation

**Figure 2:**
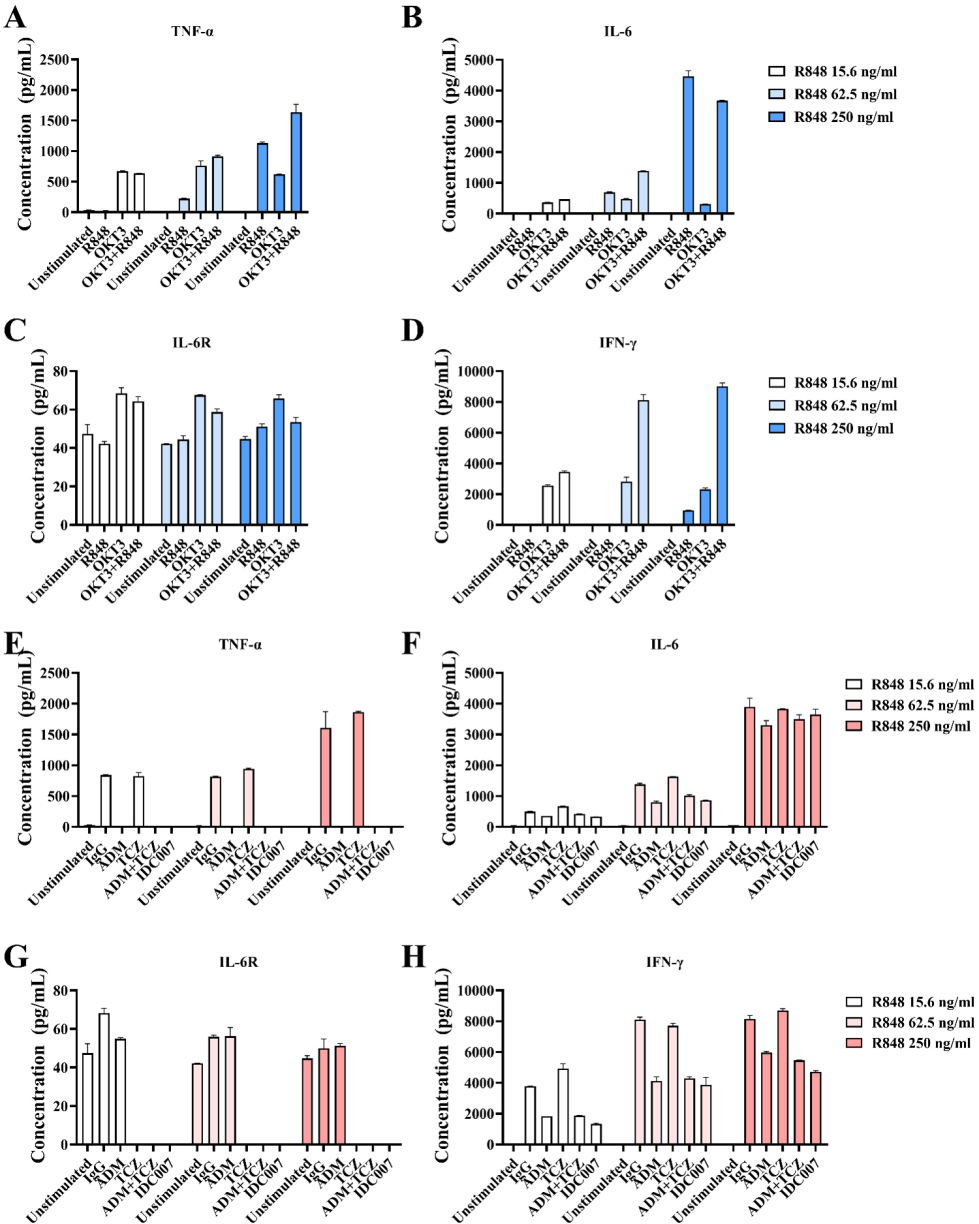
IDC007 attenuates pro-inflammatory cytokine surges in an *In Vitro* CRS model mimicking severe macrophage activation. **(A–D)** Cytokine secretion profiling following OKT3 and R848 stimulation. CRS-like conditions were induced in human PBMCs by co-treatment with OKT3 and R848, leading to robust increases in TNF-α, IL-6R, IL-6, and IFN-γ. Combined treatment with 1 μg/mL OKT3 and 250 ng/mL R848 induced approximately 2.2-fold higher IL-6 levels than TNF-α, indicating that this condition is optimal for evaluating tocilizumab efficacy. Thus, 1 μg/mL OKT3 and 250 ng/mL R848 were selected for antibody efficacy testing. **(E–H)** Modulation of cytokine secretion by antibody treatment. Under CRS-mimicking conditions, subsequent antibody treatment markedly modulated cytokine release. IDC007 effectively suppressed TNF-α and IL-6R signaling, comparable to adalimumab and tocilizumab. Although IL-6 levels remained elevated, a well-documented phenomenon resulting from the accumulation of unconsumed cytokines following potent receptor blockade, IDC007 completely abrogates its downstream signaling. Furthermore, both adalimumab and IDC007 reduced IFN-γ secretion by disrupting the cytokine-driven feedback loops between monocytes and T cells. Data are presented as the mean ± SD of 3 independent biological replicates (n = 3).

To evaluate the efficacy of IDC007 in a physiologically relevant environment, we established an *in vitro* CRS model using human peripheral blood mononuclear cells (hPBMCs). Since severe CRS involves not only T-cell activation but also the extensive engagement of myeloid cells (macrophages/monocytes) (7, 8), we stimulated hPBMCs with a combination of the anti-CD3 antibody (OKT3, 1 μg/mL) and R848 (250 ng/mL). While OKT3 treatment alone induced moderate cytokine release, the addition of R848, a potent TLR7/8 agonist known to activate monocytes (14), triggered a synergistic surge in pro-inflammatory cytokines, recapitulating the ‘cytokine storm’ observed in patients (15). Specifically, the combination treatment led to a marked elevation in TNF-α levels, reaching 1, 636 pg/mL (**Figure 2A**). While IL-6R levels remained relatively constant (**Figure 2C**), IL-6 secretion surged to 3, 674 pg/mL (**Figure 2B**), a level approximately 2.2-fold higher than that of TNF-α. Notably, the slightly lower IL-6 detection in the combined stimulation compared to R848 alone is likely attributable to increased IL-6 consumption by the massively activated T cells and counter-regulatory negative feedback loops triggered by the extreme cytokine milieu. Furthermore, to empirically validate that this combined stimulation accurately reflects the complex, multicellular hyperactivation characteristic of severe CRS, we evaluated the activation status of key immune populations. As shown in **Supplementary Figure 4**, while R848 alone induced only partial immune activation, the combination of OKT3 and R848 triggered a massive, synergistic activation across the PBMC network. Specifically, we observed a striking upregulation of the T-cell activation marker CD25 (CD3^+^CD25^+^), alongside a synergistic increase in the expression of critical co-stimulatory molecules (CD80^+^CD86^+^). These data confirm that the combined model successfully recapitulates the dynamic hyper-inflammatory crosstalk between T cells and antigen-presenting cells. Additionally, IFN-γ levels increased dramatically to 9, 015 pg/mL (**Figure 2D**), reflecting the synergistic activation of the T cell-monocyte axis. These profiles confirm the validity of this model for assessing dual-targeting efficacy against the key mediators of CRS.

In this hyper-inflammatory environment, we compared the inhibitory effects of IDC007 with those of the parental antibodies, adalimumab and tocilizumab. Treatment with IDC007 profoundly suppressed TNF-α secretion to near-baseline levels (~2 pg/mL), comparable to the efficacy of adalimumab (**Figure 2E**). Regarding soluble IL-6R, levels remained low and stable (~50 pg/mL) across all treatment groups (**Figure 2G**). Notably, while the IL-6 concentration remained elevated in the IDC007 and tocilizumab-treated groups (~3, 652 pg/mL), this accumulation is a known pharmacodynamic marker of IL-6 receptor blockade, where the antibody prevents receptor-mediated internalization and consumption of the cytokine (**Figure 2F**) (16). Despite this physical accumulation of free IL-6, the cytokine is rendered biologically inactive due to the complete masking of its target receptors.

Crucially, IDC007 treatment significantly reduced the secretion of IFN-γ (4, 724 pg/mL) compared to the IgG control (8, 152 pg/mL), exhibiting a superior suppression profile consistent with the disruption of the crosstalk between T cells and monocytes (**Figure 2H**) (11). These results demonstrate that IDC007 effectively interrupts the inflammatory feedback loop by simultaneously neutralizing TNF-α and blocking IL-6 signaling, even in the presence of intense macrophage stimulation.

Furthermore, to delineate the specific intracellular signaling mechanisms underlying this potent cytokine suppression, we evaluated the phosphorylation status of key master transcription factors in the bulk PBMC population. As shown in **Supplementary Figure 5**, the combined OKT3 and R848 stimulation markedly increased the phosphorylation of both NF-κB p65 (Ser529) (**Supplementary Figure 5A**) and STAT3 (Tyr705) (**Supplementary Figure 5B**) across the interacting immune cell network. Treatment with IDC007 effectively and simultaneously suppressed the activation of both pathways to near-baseline levels. These data provide direct, empirical evidence that IDC007 profoundly aborts the intracellular signaling cascades required for the initiation and amplification of the cytokine storm.

### 3.3 IDC007 preserves endothelial integrity by downregulating adhesion molecules and preventing VE-cadherin disruption

Elevated levels of TNF-α and IL-6 during CRS are known to compromise the endothelial barrier, leading to capillary leak syndrome and end-organ damage (17). To investigate whether the cytokine suppression achieved by IDC007 translates into endothelial protection, we treated human umbilical vein endothelial cells (HUVECs) with the conditioned media collected from the CRS model described above.

**Figure 3:**
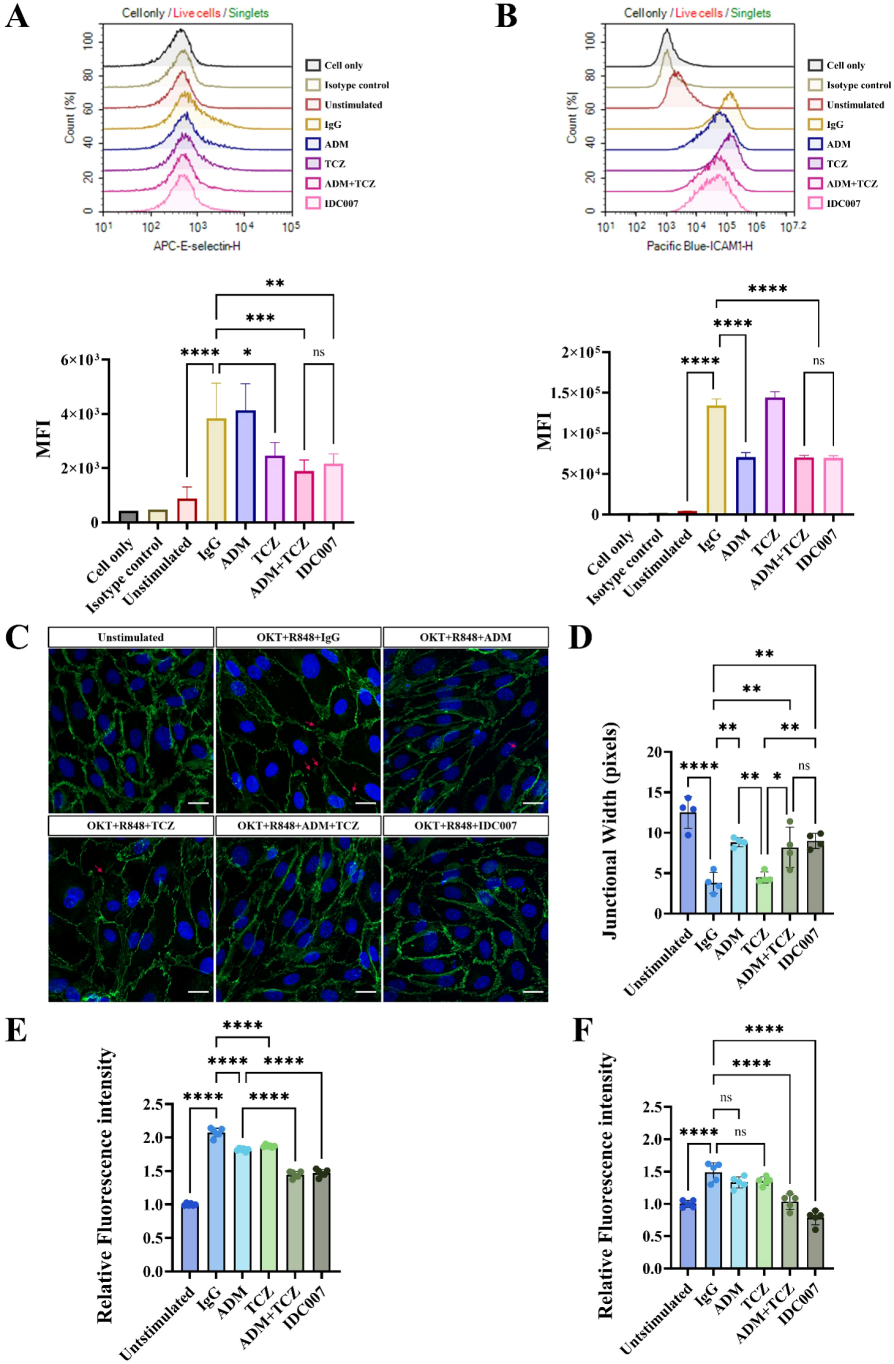
IDC007 preserves endothelial barrier integrity under simulated CRS conditions. HUVECs were treated with conditioned media from OKT3/R848-stimulated hPBMCs in the presence or absence of antibodies to assess endothelial activation, junctional integrity, and barrier function. **(A, B)** CRS-conditioned media markedly increased E-selectin and ICAM-1 expression, whereas IDC007 and the adalimumab plus tocilizumab combination reduced these adhesion molecules to approximately 50% of IgG control levels. **(C, D)** CRS-conditioned media disrupted VE-cadherin organization (red arrows denote discontinuous VE-cadherin staining at cell–cell junctions), consistent with endothelial barrier dysfunction, whereas IDC007 preserved continuous VE-cadherin junctions and restored junctional thickness to levels comparable to adalimumab-treated cells. Scale bar, 20 µm. **(E, F)** Functional assays showed that IDC007 further reduced FITC–dextran permeability beyond the effect of either adalimumab or tocilizumab monotherapy, and decreased THP-1 monocyte adhesion by about 47% compared with IgG-treated controls, demonstrating protection against CRS-associated vascular injury. Data are presented as the mean ± SD of 3 independent biological replicates (n = 3).

First, we examined the expression of endothelial adhesion molecules, E-selectin and ICAM-1, which facilitate leukocyte extravasation and perpetuate inflammation (18). Exposure to the IgG-treated CRS conditioned media dramatically upregulated the surface expression of E-selectin (~4.0 × 10^3^ MFI) and ICAM-1 (~1.5 × 10^5^ MFI) on HUVECs. However, treatment with IDC007 markedly mitigated this upregulation, reducing the expression of both markers to approximately 50% of the levels observed in the IgG control group (**Figures 3A, B**). Importantly, there was no statistical difference in the suppression levels between IDC007 and the combination treatment (adalimumab + tocilizumab). This demonstrates that the single bispecific molecule is as highly effective as the physical combination of the two parental antibodies in blunting the endothelial activation signals.

We next assessed the structural integrity of endothelial junctions by analyzing vascular endothelial (VE)-cadherin (19). Immunocytochemical analysis revealed that the CRS environment caused a severe disruption of VE-cadherin junctions, characterized by fragmented staining patterns and a significant reduction in junctional thickness (~4 pixels). In contrast, IDC007 prevented this loss, preserving continuous linear junctions with a thickness (~9 pixels) comparable to that of the unstimulated control (**Figures 3C, D**).

Interestingly, comparative analysis of the monotherapies revealed distinct mechanistic contributions: adalimumab played a dominant role in suppressing ICAM-1 upregulation and preserving VE-cadherin architectural integrity, whereas tocilizumab preferentially inhibited the expression of E-selectin. These findings align with the established leukocyte adhesion cascade model (18), wherein TNF-α acts as a primary driver of endothelial structural disruption and firm adhesion, while IL-6 trans-signaling facilitates endothelial activation and selectin-mediated leukocyte rolling. Consequently, IDC007 exerts a synergistic therapeutic effect by simultaneously intercepting these complementary pathways of vascular injury.

To confirm the functional consequence of these molecular changes, we performed a vascular permeability assay using FITC-dextran and a monocyte adhesion assay. Consistent with the loss of VE-cadherin, the CRS conditioned media increased endothelial permeability by 2-fold compared to baseline. IDC007 treatment significantly attenuated this increase, reducing permeability to levels significantly lower than IgG control (~1.5-fold relative increase), which was superior to monotherapy with adalimumab or tocilizumab alone (**Figure 3E**). Furthermore, IDC007 effectively inhibited the adhesion of THP-1 monocytes to the activated endothelial layer, reducing adhesion by approximately 47% compared to the IgG control, thereby restoring it to near-baseline levels (**Figure 3F**). Collectively, these data demonstrate that IDC007 protects against CRS-associated vascular injury by maintaining endothelial junctional integrity and limiting inflammatory cell infiltration.

### 3.4 Enhanced efficacy of dual TNF-α and IL-6R blockade in ameliorating CRS pathology in a syngeneic mouse model

**Figure 4:**
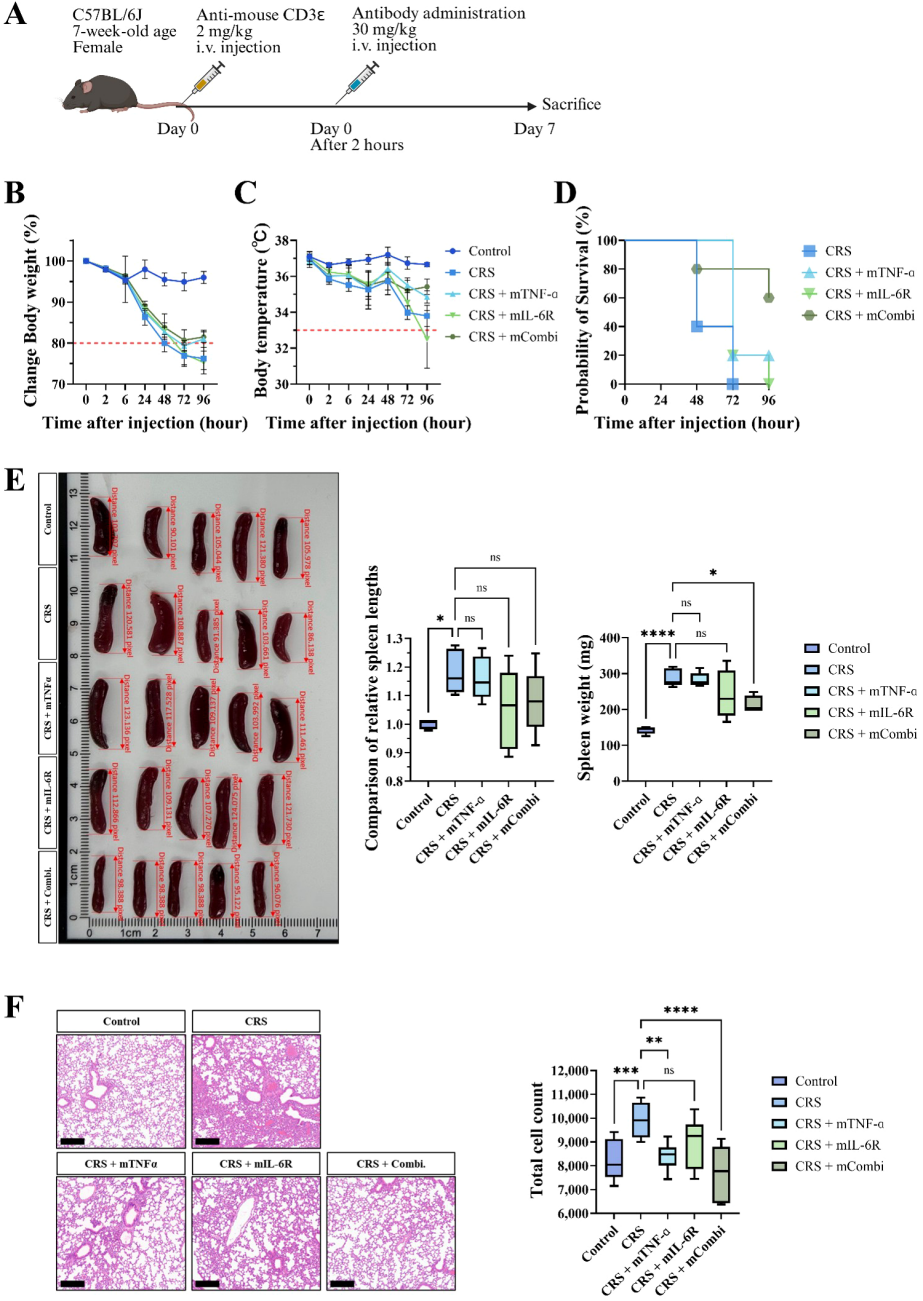
Therapeutic efficacy of dual TNF-α and IL-6R blockade in a syngeneic mouse model of CRS. C57BL/6J mice were intravenously injected with anti-CD3ϵ antibody (2 mg/kg) to induce CRS. Therapeutic antibodies (30 mg/kg of mTNF-α, mIL-6R, or a combination of both) were administered intravenously 2 hours post-induction. **(A)** Schematic representation of the syngeneic CRS mouse model with antibody administration 2 hours post-induction. Schematic illustration created with BioRender. **(B)** Percentage change in body weight was monitored over 96 hours. **(C)** Rectal body temperature was measured at the indicated time points to assess CRS-associated hypothermia. **(D)** Survival rates were analyzed. **(E)** Assessment of splenomegaly as a marker of inflammation. Images of spleens harvested at the endpoint are shown (left). Quantitative analysis of relative spleen lengths (middle) and spleen weights (right) is presented as box-and-whisker plots (min to max). **(F)** Representative H&E-stained lung tissue sections showing inflammatory cell infiltration and alveolar damage. The severity of lung inflammation was quantified by counting the total number of nucleated cells per FOV (right). Note that all organ evaluations and histological analyses were performed strictly on tissues collected from surviving mice euthanized at the scheduled day 7 endpoint. Scale bar, 200 µm (original magnification, 20×). The red dashed lines indicate a 20% loss of initial body weight and a body temperature of 33 °C. Data in **(B)** and **(C)** are presented as mean ± SD. Data in **(E, F)** are presented as box-and-whisker plots (min to max) to illustrate the distribution of tissue damage. Statistical significance was determined using one-way ANOVA followed by Dunnett’s multiple comparisons test compared to the CRS group (n = 5 mice per group). *p < 0.05, **p < 0.01, ***p < 0.001, ****p < 0.0001; ns, not significant.

To evaluate the therapeutic potential of blocking both TNF-α and IL-6R, we first utilized an anti-CD3ϵ-antibody-induced CRS syngeneic mouse model (12). C57BL/6J mice were treated with surrogate antibodies targeting mouse TNF-α, mouse IL-6R, or a combination of both (mCombi) 2 hours after CRS induction (**Figure 4**). To thoroughly evaluate the therapeutic window, we compared a prophylactic setting (intervention concurrently at 0 hours) with a therapeutic setting (delayed intervention at 2 hours). The 2-hour delay was specifically chosen because it is a well-established characteristic of anti-CD3-induced CRS models that the critical upstream initiator TNF-α typically peaks within 1 to 2 hours post-induction, mimicking a clinical scenario where intervention occurs immediately following the acute onset of the cytokine storm. The CRS-induced group exhibited severe physiological deterioration, including rapid weight loss and significant hypothermia. While monotherapy with anti-mTNF-α or anti-mIL-6R provided partial relief, the simultaneous blockade of both pathways (mCombi) resulted in attenuated weight loss and hypothermia (**Figures 4B, C**). Consistently, the mCombi group showed an improvement in survival rates compared to the monotherapy and vehicle-treated groups (**Figure 4D**). Organ inflammation was further assessed by examining splenomegaly and lung pathology. The mCombi treatment significantly reduced spleen weight, which was enlarged due to immune activation in the CRS group (**Figure 4E**). Furthermore, histological analysis of lung tissue revealed that the combination therapy effectively inhibited inflammatory cell infiltration and alveolar damage compared to the CRS control (**Figure 4F**).

**Figure 5:**
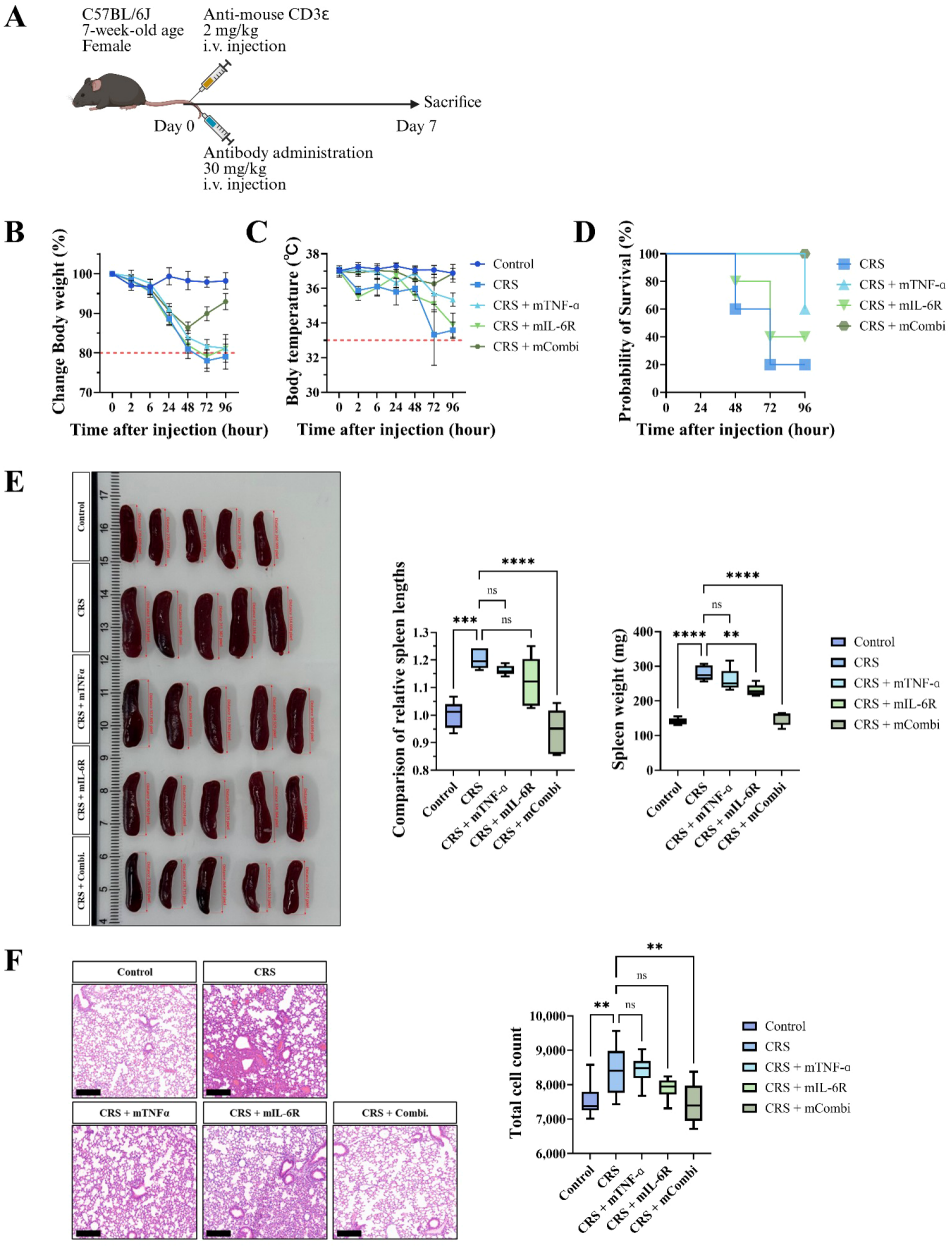
Efficacy of simultaneous dual TNF-α and IL-6R blockade in a syngeneic mouse model of CRS. C57BL/6J mice were intravenously injected with anti-CD3ϵ antibody (2 mg/kg) to induce CRS. Therapeutic antibodies (30 mg/kg of mTNF-α, mIL-6R, or a combination of both) were administered intravenously simultaneously with CRS induction. **(A)** Schematic representation of the syngeneic CRS mouse model with simultaneous antibody administration. Schematic illustration created with BioRender. **(B)** Percentage change in body weight was monitored over 96 hours. **(C)** Rectal body temperature was measured to assess the prevention of hypothermia. **(D)** Survival rates were analyzed. **(E)** Assessment of splenomegaly. Images of spleen (left), relative spleen lengths (middle), and spleen weights (right) are shown. **(F)** Histopathological analysis of lung injury. Representative H&E-stained lung sections (left) show inflammatory infiltration. The severity of lung inflammation was quantified by counting the total number of nucleated cells per FOV (right). Note that all organ evaluations and histological analyses were performed strictly on tissues collected from surviving mice euthanized at the scheduled day 7 endpoint. Scale bar, 200 µm (original magnification, 20×). The red dashed lines indicate a 20% loss of initial body weight and a body temperature of 33 °C. Data in **(B, C)** are presented as mean ± SD. Data in **(E, F)** are presented as box-and-whisker plots (min to max) to illustrate the distribution of tissue damage. Statistical significance was determined using one-way ANOVA followed by Dunnett’s multiple comparisons test compared to the CRS group (n = 5 mice per group). **p < 0.01, ***p < 0.001, ****p < 0.0001; ns, not significant.

We next investigated the rapid intervention efficacy and immunological mechanisms of dual blockade by administering the antibodies simultaneously with CRS induction (**Figure 5**). In this setting, the mCombi group demonstrated complete protection against CRS-associated mortality (100% survival) and prevented significant changes in body weight and temperature (**Figures 5B–D**). To elucidate the impact of dual blockade on immune cell dynamics, we analyzed the population of T cells and myeloid cells in peripheral blood (**Supplementary Figures 2A, B**). CRS induction typically leads to T-cell lymphopenia due to excessive immune response (20, 21). As expected, the CRS group showed a sharp decline in CD3^+^ T cells. However, mCombi treatment significantly restored these T-cell populations to levels comparable to the healthy control group (**Supplementary Figure 2A**). Conversely, the expansion of inflammatory myeloid populations, including CD11b^+^, CD14^+^ monocytes, and CD11b^+^ Gr-1^+^ myeloid-derived suppressor cells (MDSCs), was significantly suppressed in the mCombi group (**Supplementary Figure 2B**). These results suggest that dual blockade not only attenuates inflammation but also helps restore immune homeostasis. Consistent with these findings, the simultaneous regimen also effectively prevented splenomegaly (**Figure 5E**) and significantly reduced the infiltration of nucleated cells into the lung tissue, as quantified by histological analysis (**Figure 5F**). Taken together, these findings collectively underscore the superior therapeutic potential of a dual-targeting strategy over conventional monotherapies in managing CRS. By simultaneously inhibiting both TNF-α and IL-6R signaling pathways, the combination therapy not only abrogated the hyper-inflammatory cytokine cascade but also effectively prevented organ damage and restored immune homeostasis. Moreover, the marked difference in survival outcomes between the therapeutic setting (partial rescue, **Figure 4D**) and the simultaneous intervention (100% survival, **Figure 5D**) highlights the critical importance of rapid intervention. Our data suggest that prompt dual blockade can effectively attenuate the initial rapid elevation of inflammatory mediators, thereby preventing the onset of irreversible physiological deterioration and facilitating a complete recovery from the inflammatory crisis.

### 3.5 Superior therapeutic efficacy of the bispecific antibody IDC007 in a humanized CRS model

**Figure 6:**
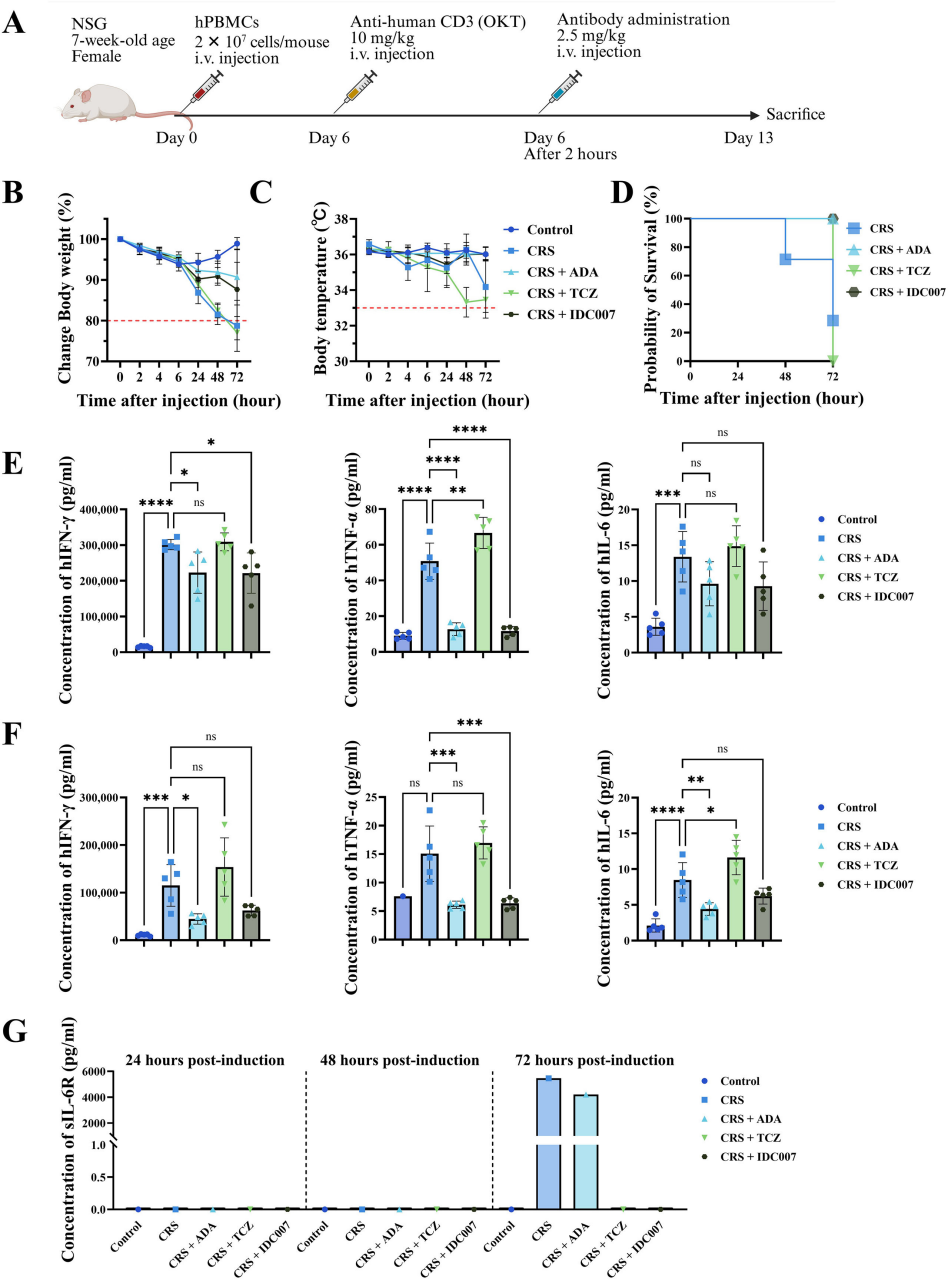
Comparison of therapeutic efficacy between IDC007 and conventional monotherapies in a humanized CRS mouse model. NSG mice were intravenously engrafted with hPBMCs (2 × 10^7^ cells/mouse) to establish a humanized model. One-week post-engraftment, CRS was induced by intravenous injection of OKT3. Therapeutic antibodies (2.5 mg/kg of Adalimumab, Tocilizumab, or IDC007) were administered intravenously, 2 hours post-induction. **(A)** Schematic representation of the humanized CRS mouse model with antibody administration 2 hours post-induction. Schematic illustration created with BioRender. **(B)** Percentage change in body weight and **(C)** rectal body temperature was monitored to evaluate physiological recovery from CRS. **(D)** Survival rates were analyzed. **(E, F)** Serum concentrations of human pro-inflammatory cytokines (hIFN-γ, hTNF-α, and hIL-6) were measured at **(E)** 6 hours and **(F)** 24 hours post-induction using multiplex assays. **(G)** Serum concentrations of sIL-6R were assessed at 24, 48, and 72 hours (from left to right) to evaluate target neutralization. The red dashed lines indicate a 20% loss of initial body weight and a body temperature of 33 °C. Data in **(B, C, E, F)** are presented as mean ± SD. Statistical significance was determined using one-way ANOVA followed by Dunnett’s multiple comparisons test compared to the CRS group (n = 5 mice per group). Data in **(G)** represent sIL-6R concentrations measured from serum samples pooled by group. *p < 0.05, **p < 0.01, ***p < 0.001, ****p < 0.0001; ns, not significant.

To validate the clinical relevance of our findings, we evaluated the therapeutic efficacy of IDC007, a bispecific antibody targeting human TNF-α and IL-6R, in a humanized CRS mouse model (13). NSG mice engrafted with hPBMCs were induced with CRS using OKT3 and treated 2 hours later with IDC007, adalimumab, or tocilizumab (**Figure 6**). While prophylactic administration yielded optimal protection in the syngeneic model, clinical management of CAR-T-induced CRS strictly relies on therapeutic intervention after symptom onset to avoid blunting anti-tumor efficacy. Therefore, to rigorously evaluate the translational medical significance of IDC007 as a clinical rescue therapy, we deliberately applied only the stricter 2-hour delayed therapeutic setting in this humanized model. Physiologically, IDC007 and adalimumab demonstrated superior protective effects compared to tocilizumab. While tocilizumab showed limited efficacy in preventing weight loss and hypothermia, IDC007-treated mice effectively maintained conditions similar to the non-induced Control group (**Figures 6B, C**). Consistent with these physiological parameters, both IDC007 and adalimumab treatments resulted in a 100% survival rate, whereas the tocilizumab group exhibited significant mortality, comparable to the untreated CRS group (**Figure 6D**). We further analyzed the serum concentrations of key human pro-inflammatory cytokines to verify the rapid establishment of the model (13, 17). Confirming the successful and acute induction of CRS, levels of hIFN-γ, hTNF-α, and hIL-6 in the untreated CRS group increased dramatically as early as 6 hours, and remained elevated at 24 hours post-induction compared to the Control group. Both adalimumab and IDC007 treatments significantly suppressed the levels of all three major cytokines at both time points, indicating potent amelioration of the cytokine storm (**Figures 6E, F**). In contrast, tocilizumab failed to reduce these inflammatory mediators, instead showing a marked accumulation of serum hIL-6 (**Figure 6F** right) (16), likely due to receptor blockade preventing cytokine consumption. Additionally, we monitored the levels of sIL-6R, one of the therapeutic targets of IDC007 and a key mediator of IL-6 trans-signaling (22). While sIL-6R levels remained elevated in the CRS and adalimumab groups, particularly at 72 hours, sIL-6R was virtually undetectable in both the tocilizumab and IDC007-treated groups at 24, 48, and 72 hours (**Figure 6G**). This suggests that IDC007 effectively neutralizes the target via complete epitope masking, thereby preventing the amplification of inflammatory signals via the trans-signaling pathway. Collectively, IDC007 effectively reduced the detectable free levels of the target proteins associated with both tocilizumab and adalimumab. Notably, IDC007 combined the physiological efficacy observed with adalimumab with the robust target engagement of sIL-6R (evidenced by the depletion of measurable free sIL-6R), effectively blocking the trans-signaling pathway without the accompanying IL-6 accumulation observed with tocilizumab. These *in vivo* results demonstrate that IDC007 overcomes the limitations of single-target therapies, offering a comprehensive therapeutic strategy for managing severe CRS.

## 4 Discussion

CRS remains a significant clinical bottleneck in the application of potent T-cell-engaging immunotherapies (23, 24). While IL-6 receptor blockade with tocilizumab is the current standard of care, it often fails to control severe, rapid-onset CRS or prevent neurotoxicity, highlighting the critical need for broader upstream inhibition (24). In this study, we developed and characterized IDC007, a bispecific antibody simultaneously targeting TNF-α and IL-6R and demonstrated its superior therapeutic efficacy in mitigating CRS pathology through multi-layered mechanisms involving immune modulation and endothelial protection (25). Furthermore, while a recent study reported a bispecific antibody targeting TNF-α and IL-6R, IDC007 features distinct structural and translational innovations. The previously reported molecule relies on an scFv-scFv fusion format containing a polyhistidine tag (26, 27). In contrast, IDC007 is designed as an IgG-scFv appended format retaining the intact Fc region. The presence of the Fc domain enables FcRn-mediated recycling *in vivo*, providing an extended serum half-life and allowing for sustained therapeutic efficacy even at lower doses. Importantly, IDC007 is completely devoid of a His-tag, an immunogenic sequence strictly avoided in FDA-approved clinical therapeutics, ensuring a highly translatable, clinical-grade safety profile.

Our *in vitro* findings using the OKT3/R848-stimulated PBMC model provide persuasive evidence for the necessity of dual blockade. This model recapitulates the severe macrophage activation observed in high-grade CRS (8), where single-target inhibition often proves insufficient. In this hyper-inflammatory environment, IDC007 not only neutralized its primary targets but also effectively orchestrated the interruption of pathogenic crosstalk between T cells and monocytes (7, 8, 25). By inhibiting the upstream initiator TNF-α, IDC007 suppressed the overall inflammatory drive, leading to a consequent reduction in the secretion of downstream mediators such as IFN-γ (**Figure 2H**). Furthermore, we elucidated the direct protective impact of IDC007 on the vascular endothelium. Given that severe CRS is characterized by capillary leak syndrome driven by endothelial activation (17), our observation that IDC007 preserved the structural integrity of VE-cadherin junctions (19) and downregulated the expression of adhesion molecules E-selectin and ICAM-1 is particularly significant (**Figures 3A–F**) (18). These results suggest that IDC007 targets the fundamental mechanism of organ damage, vascular dysfunction, more effectively than current monotherapies.

To rigorously validate the therapeutic potential of IDC007, we employed a humanized *in vivo* model. In this setting, IDC007 demonstrated remarkable efficacy, achieving 100% survival, whereas tocilizumab monotherapy failed to prevent mortality (**Figure 6D**). Crucially, the ability of IDC007 to successfully rescue humanized mice even when administered 2 hours post-induction, a time point when the TNF-α-driven cascade is already biologically established, highlights its robust translational potential as an interventional rescue therapeutic, rather than merely a prophylactic agent. Beyond survival benefits, our data highlights a distinct mechanistic advantage of IDC007 regarding cytokine kinetics. A well-documented limitation of tocilizumab is the accumulation of serum IL-6 following receptor blockade (16, 28), which may potentially divert the cytokine to alternative signaling pathways or delay the resolution of inflammation. In contrast, IDC007 treatment did not result in a concurrent accumulation of IL-6 (**Figure 6F**). Moreover, IDC007 achieved comprehensive target engagement, as evidenced by the complete neutralization of free sIL-6R, the key mediator of the pro-inflammatory trans-signaling pathway (**Figure 6G**) (28, 29). This suggests that, by reducing the bioavailability of sIL-6R, IDC007 may mechanistically prevent the formation of the pathogenic IL-6/sIL-6R complex or prevent the feedback accumulation of inflammatory mediators, thereby overcoming a significant pharmacokinetic limitation of existing anti-IL-6R therapies. However, careful analysis of the humanized model results is required due to species-specific limitations. It is well established that while human IL-6 exhibits cross-reactivity with the murine IL-6 receptor (30), enabling it to induce inflammatory signaling in murine host tissues, therapeutic antibodies targeting human IL-6R (such as tocilizumab) do not cross-react with the murine receptor (31). Accordingly, in the tocilizumab-treated group, the excessive release of human IL-6 likely activated the unblocked murine IL-6Rs on host tissues, driving lethal inflammation via the IL-6 signaling pathway. The success of IDC007 in this model, therefore, is largely attributable to the neutralization of human TNF-α. By blocking this upstream master regulator released by the engrafted human T cells, IDC007 effectively abrogated the initiation of the cytokine storm at its source before the murine IL-6 pathway could become the dominant driver. This finding underscores the critical importance of upstream TNF-α blockade but also highlights the inability of the humanized model to fully evaluate the synergistic contribution of the anti-IL-6R arm of IDC007. Furthermore, while the syngeneic model effectively validates the therapeutic rationale of dual blockade, it relied on a combination of monoclonal antibodies rather than a surrogate bispecific molecule. Therefore, the *in vivo* data presented herein should be interpreted strictly as a proof-of-concept for the dual-targeting strategy, whereas definitively proving the unique pharmacokinetic and pharmacodynamic advantages of the bispecific format requires future studies utilizing humanized double knock-in mice or surrogate bispecific antibodies.

**Figure 7:**
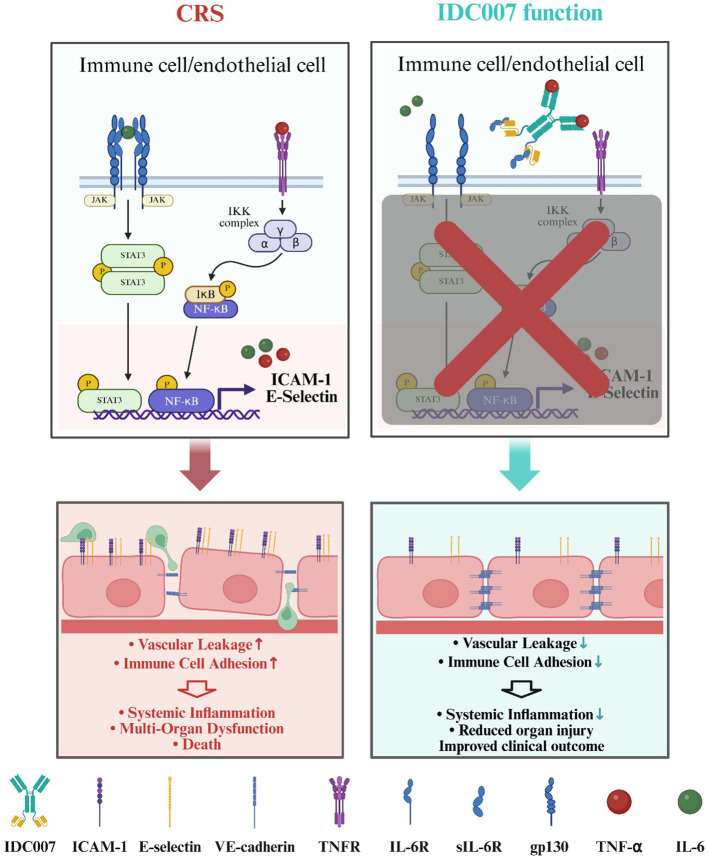
Schematic of the molecular and cellular mechanisms underlying the therapeutic efficacy of IDC007 in severe CRS. (Left) Under severe CRS conditions, the hyper-inflammatory milieu is driven by upstream TNF-α activating the canonical NF-κB pathway via the IKK complex, alongside the IL-6/sIL-6R complex binding to gp130, which triggers trans-signaling and subsequent STAT3 phosphorylation via JAK. This synergistic intracellular signaling leads to severe endothelial dysfunction, characterized by the nuclear transcription and upregulation of adhesion molecules (ICAM-1 and E-selectin) and the disruption of VE-cadherin junctions. These alterations result in massive vascular leakage, increased immune cell adhesion, and subsequent lethal multi-organ damage. (Right) IDC007 effectively dismantles this pathological crosstalk. By simultaneously neutralizing TNF-α and blocking sIL-6R-mediated signaling, IDC007 prevents receptor engagement and effectively inhibits the downstream NF-κB and STAT3 signaling cascades. This dual blockade directly preserves endothelial barrier integrity by maintaining the stable architecture of VE-cadherin and suppressing ICAM-1/E-selectin expression, ultimately mitigating severe immune-mediated inflammation and providing robust protection against CRS-induced organ damage. This figure was created using BioRender.

Consequently, as presented earlier, to overcome this species cross-reactivity limitation and fully validate the dual-targeting mechanism in an intact immune system, we utilized syngeneic C57BL/6J models with surrogate antibodies against murine TNF-α and IL-6R. In a therapeutic setting where antibodies were administered 2 hours after CRS induction, the dual blockade successfully rescued mice from inflammation, attenuating physiological deterioration (weight loss and hypothermia) compared to monotherapies (**Figure 4**). Furthermore, when administered simultaneously with CRS induction, the dual blockade provided complete protection and maintained T-cell homeostasis while suppressing inflammatory myeloid expansion (**Supplementary Figure 2**). Recent single-cell RNA sequencing (scRNA-seq) studies of CRS, particularly in the context of CAR-T cell therapy, have elucidated that the hyper-inflammatory network is predominantly driven by activated bystander monocytes and macrophages (7, 8, 32). In this complex immune network, TNF-α acts as a critical upstream initiator that robustly activates the nuclear factor kappa-light-chain-enhancer of activated B cells (NF-κB) signaling pathway, leading to the widespread transcription of downstream pro-inflammatory mediators, including large quantities of IL-6 (33). Subsequently, IL-6 functions as a systemic amplifier by signaling through the Janus kinase/signal transducer and activator of transcription 3 (STAT3) pathway, which exacerbates endothelial activation, acute-phase responses, and further inflammatory myeloid cell recruitment. Crucially, NF-κB and STAT3 are known to tightly collaborate to form a pathogenic positive feedback loop, where NF-κB-induced cytokines drive STAT3 activation, which in turn prolongs and amplifies NF-κB-dependent inflammatory cascades (34, 35). To validate this specific pathogenic disease circuit *in vivo*, our analysis of a publicly available CRS scRNA-seq dataset (GSE198868) confirmed that T-cell engager therapies robustly drive the hyperactivation of both NF-κB (RELA) and STAT3 pathways, predominantly within the myeloid and endothelial compartments (**Supplementary Figures 6A**, **B**). Building upon this transcriptomic baseline, our targeted phospho-flow cytometry provides highly complementary orthogonal validation at the protein level (**Supplementary Figure 5**). This divergence in pathway activation across cellular compartments likely stems from differences in temporal dynamics and experimental modalities. Phospho-flow cytometry captures acute, transient protein phosphorylation at an early 6-hour time point, reflecting the role of immune cells as primary cytokine sources. In contrast, scRNA-seq analysis infers cumulative transcriptomic changes based on downstream gene expression. Over time, activated immune cells typically undergo negative feedback or signal desensitization, resulting in stabilized transcriptomic profiles. Conversely, the vascular endothelium serves as a continuous structural target for circulating cytokines, leading to sustained transcriptional upregulation of the NF-κB and STAT3 axes (5, 17, 22). Thus, these orthogonal approaches provide complementary spatiotemporal insights, mapping CRS progression from acute immune activation to sustained endothelial dysfunction. As directly evidenced by these functional results, IDC007 fundamentally dismantles this synergistic NF-κB/STAT3 crosstalk across the interacting immune cell populations. By simultaneously suppressing the phosphorylation of NF-κB p65 (Ser529) and STAT3 (Tyr705), IDC007 concurrently abrogates both the upstream initiation and the downstream amplification phases. This systemic reprogramming of hyperactivated monocyte/macrophage populations facilitates the rapid restoration of immune homeostasis without the need for prolonged immune cell depletion. This early intervention translated into robust organ protection, evidenced by the prevention of splenomegaly and a marked reduction in pulmonary cellular infiltration (**Figures 5E, F**). Overall, as comprehensively summarized in our mechanistic model (**Figure 7**), the simultaneous neutralization of TNF-α and masking of sIL-6R by IDC007 synergistically aborts both the canonical IKK/NF-κB and JAK/STAT3 trans-signaling cascades (29). This dual blockade not only suppresses hypercytokinemia but fundamentally preserves endothelial barrier integrity by maintaining VE-cadherin architecture and downregulating adhesion molecules (ICAM-1 and E-selectin), ultimately preventing lethal multi-organ damage. These syngeneic model data complement what the humanized model could only partially suggest, showing that simultaneous blockade of TNF-α and IL-6R provides a synergistic therapeutic benefit superior to single-pathway inhibition. Importantly, the striking difference in survival rates between the therapeutic (2 hours post-induction) and simultaneous settings clearly demonstrates that the timing of intervention is determinative. The cytokine storm operates as an exponential amplification loop; once the inflammatory cascade reaches a critical threshold, reversing the consequent multi-organ damage becomes increasingly challenging (25). The complete protection achieved by simultaneous administration indicates that early dual blockade of both the TNF-α initiator and IL-6R effector pathways provides a profound clinical advantage, neutralizing the storm before irreversible vascular and tissue damage can occur.

Despite the promising therapeutic efficacy demonstrated in our acute CRS models, this study has limitations that warrant further investigation. First, while our models successfully recapitulated the acute macrophage-driven cytokine storm, they do not involve CAR-T cells. Future studies utilizing CAR-T-induced CRS models in tumor-bearing mice will be essential to definitively confirm that IDC007 mitigates toxicity without compromising the anti-tumor efficacy and *in vivo* persistence of engineered T cells. Second, the current study lacks comprehensive *in vivo* systemic safety data, such as evaluation of potential immunosuppression, cytokine rebound, and standard toxicity assessments (e.g., hematological parameters and full histopathological examination of major organs). Conducting extensive GLP-compliant toxicity profiling to thoroughly evaluate long-term immune homeostasis falls beyond the scope of this initial proof-of-concept study and will be rigorously conducted in forthcoming IND-enabling studies. Third, given the central pathogenic roles of TNF-α and IL-6 in various immune and autoinflammatory conditions, evaluating the broader therapeutic potential of this dual-targeting strategy in other cytokine-driven disease models represents a highly translatable avenue for our ongoing research. Finally, our *in vivo* sIL-6R quantification was limited to measuring free receptors due to epitope competition in the current ELISA format; future studies utilizing non-competing capture antibodies are required to comprehensively evaluate total sIL-6R kinetics.

## Data Availability

The datasets presented in this article are not readily available because they contain proprietary and commercial information. Requests to access the datasets should be directed to the corresponding authors, and may be subject to a non-disclosure agreement (NDA) and institutional approval.

## Glossary

BiTE: Bispecific T-cell engager
CAR: Chimeric antigen receptor
CD3: Cluster of differentiation 3
CRS: Cytokine release syndrome
DAPI: 4′, 6-diamidino-2-phenylindole
DMSO: Dimethyl sulfoxide
DPBS: Dulbecco’s phosphate-buffered saline
EC: Endothelial cells
E-selectin: Endothelial selectin
EC50: Half-maximal effective concentration
ELISA: Enzyme-linked immunosorbent assay
FACS: Fluorescence-activated cell sorting
FBS: Fetal bovine serum
FDR: False discovery rate
FOV: Field of view
H&E: Hematoxylin and eosin
hPBMCs: Human peripheral blood mononuclear cells
HUVEC: Human umbilical vein endothelial cells
IC50: Half-maximal inhibitory concentration
ICAM-1: Intercellular adhesion molecule-1
ICC: Immunocytochemistry
IFN-γ: Interferon-gamma
IgG: Immunoglobulin G
IL-6: Interleukin-6
IL-6R: Interleukin-6 receptor
KD: Equilibrium dissociation constant
Mac: Macrophages
MDSC: Myeloid-derived suppressor cells
MFI: Mean fluorescence intensity
Mono: Monocytes
Neu: Neutrophils
NF-κB: Nuclear factor kappa-light-chain-enhancer of activated B cells
NSG: NOD scid gamma
PBS: Phosphate-buffered saline
PFA: Paraformaldehyde
PoC: Proof-of-concept
scFv: Single-chain variable fragment
SDS-PAGE: Sodium dodecyl sulfate-polyacrylamide gel electrophoresis
SE-FPLC: Size-exclusion fast protein liquid chromatography
sIL-6R: Soluble interleukin-6 receptor
SPR: Surface plasmon resonance
STAT3: Signal transducer and activator of transcription 3
TDB: T-cell-dependent bispecific antibody
TF: Transcription factor
TLR: Toll-like receptor
TNF-α: Tumor necrosis factor-alpha
TNFR: Tumor necrosis factor receptor
VE-cadherin: Vascular endothelial cadherin.
