# Supplementary material for: Dual Inhibition of TNF-α and IL-6R mitigates cytokine release syndrome via protection of endothelial integrity and reduction of organ damage in mouse models
Source: Front Immunol. 2026 Jul 10;17:1848290. doi: 10.3389/fimmu.2026.1848290 (PMC13398377; doi:10.3389/fimmu.2026.1848290)
Supplement: Supplementary file 1 [file DataSheet1.docx]

1. Supplementary Material
   1. Materials and methods
      1. ***Antibody Generation and Purification***To generate the bispecific antibody IDC007, ExpiCHO-S cells (Thermo Fisher Scientific) were co-transfected with plasmids encoding the adalimumab light chain and a fusion heavy chain, comprising the adalimumab heavy chain linked to a tocilizumab-derived scFv. Following expression, the antibody was purified from the culture supernatant using a Protein A affinity column (KANEKA KanCapA, KPA02-B) connected to a peristaltic pump (Longer, BT100-1L). Bound antibodies were eluted with 20 mM citrate buffer containing 1 M urea (pH 4.2) and subsequently formulated in Dulbecco's phosphate-buffered saline (DPBS, pH 7.4) for further analysis and *in vivo* studies.
      2. ***Antibody Characterization and Quality Control***
         The physicochemical properties and structural integrity of the purified IDC007 were assessed using standard biochemical analyses. Protein concentration was determined by measuring absorbance at 280 nm using a spectrophotometer (DS-11, DeNovix), resulting in a final concentration of 3.34 mg/mL and a production yield of approximately 285 mg/L. Purity and molecular weight were analyzed by SDS-PAGE under both reducing and non-reducing conditions. For the final product used in this study, SDS-PAGE revealed a single intact band at approximately 198 kDa under non-reducing conditions, and two distinct bands corresponding to the heavy chain-scFv fusion (~76 kDa) and light chain (~23 kDa) under reducing conditions. The monomeric purity was further confirmed to be >95.0% by SE-FPLC.
      3. ***Flow Cytometric Analysis of Peripheral Immune Cells***
         For *in vivo* experiments (immune cell populations), whole blood was collected from C57BL/6J mice following CRS induction and treatment administration at the time of sacrifice. Blood samples were collected into EDTA-treated tubes (BD Biosciences). Red blood cells were removed using ACK lysing buffer (Gibco) according to the manufacturer’s instructions. The resulting cells were stained with a comprehensive panel of fluorochrome-conjugated antibodies or their corresponding isotype controls to ensure specific identification. For T-cell characterization, the cells were stained with APC anti-mouse CD3ε (BioLegend, 100311), PE anti-mouse CD4 (BioLegend, 100408), and eFluor™ 450 anti-mouse CD8a (Invitrogen, 48-0081-82). Myeloid and monocytic populations were identified using FITC anti-mouse/human CD11b (BioLegend, 101205), APC anti-mouse CD14 (BioLegend, 123312), and PE anti-mouse Ly-6G/Ly-6C (Gr-1) (BioLegend, 108408). The following isotype-matched controls were employed to account for non-specific binding: APC Armenian Hamster IgG (BioLegend, 400912), PE Rat IgG2b, κ (BioLegend, 400608), eFluor™ 450 Rat IgG2a, κ (Invitrogen, 48-4321-82), FITC Rat IgG2b, κ (BioLegend, 400606), and APC Rat IgG2a, κ (BioLegend, 400512). Data acquisition was performed on a flow cytometer (Agilent, 2010064AA). During analysis, nucleated cells were first plotted using forward scatter area and forward scatter height to gate for single cells and exclude doublets. Following singlet gating, fluorescence compensation was applied to correct for spectral overlap between channels. Targeted immune populations were identified based on the expression of surface markers, and all data were processed using the instrument's software.
      4. ***TNF-α Neutralization Assay***TNF-α neutralization activity was assessed using Actinomycin D-sensitized WEHI-13VAR cells in assay medium consisting of RPMI 1640 supplemented with 2% (v/v) FBS. Serially diluted Humira, PBP1502, or human IgG (final concentrations 0.293–150 µg/mL; 100 μL/well) were added to 96-well plates, followed by human TNF-α (NIBSC, 12/154; final 24.84 IU/mL, 50 μL/well). Cells (5 × 10⁴/well in assay medium containing 2 μg/mL Actinomycin D, Sigma, A9415) were seeded in triplicate and incubated for 20 h at 37°C, 5% CO₂. CellTiter 96® AQueous One Solution MTS (Promega, G5430; 20 μL/well) was added, plates incubated for an additional 5 h, and absorbance measured at 490 nm. EC₅₀ values were calculated from dose-response curves using four-parameter logistic regression in GraphPad Prism.
      5. ***IL-6R Neutralizing Antibody Assay***
         IL-6R neutralizing activity was assessed using IL-6 Bioassay Cells (Promega, J3025). Frozen cells were rapidly thawed in a 37°C water bath for 2 min and seeded at 2.5 × 10⁴ cells/well (50 μL/well in Bioassay Medium: RPMI 1640 + 10% FBS) into white, flat-bottom 96-well assay plates (Corning, 3917). TCZ (Selleckchem), TCZ (IDC) or human IgG (final concentrations 0.007621–50 µg/mL) were added to seeded cells and pre-incubated for 20 min at 37°C, 5% CO₂. Recombinant human IL-6 (PeproTech, 200-06; final 120 ng/mL) was then added, and cells were incubated for an additional 6 h at 37°C, 5% CO₂. Bio-Glo™ Luciferase Assay Reagent (Promega, G7940; 75 μL/well) was added, plates incubated for 10 min at room temperature, and luminescence measured using a plate luminometer (1 s integration/well). IC₅₀ values were calculated from dose-response curves using four-parameter logistic regression in GraphPad Prism.
      6. ***Flow Cytometric Analysis of Immune Cell Activation***

OKT3 (BioXCell, BE0001-2) was diluted to 1 μg/mL in PBS and coated onto 24-well plates (1 mL/well) by incubation at 37°C for 1 h. The plates were washed twice with PBS. Human PBMCs were seeded at 2 × 10⁶ cells/well in RPMI 1640 (Gibco, 11875-093) supplemented with 10% (v/v) FBS and 1× antibiotic-antimycotic. R848 was added to a final concentration of 250 ng/mL, reaching a total volume of 1 mL/well. Following a 24-h incubation at 37°C with 5% CO₂, the cells were harvested for flow cytometric analysis. To evaluate distinct targeted immune populations, the cells were stained in separate staining panels. For T-cell activation, cells were stained with Brilliant Violet 421™ anti-human CD3 (BioLegend, 317344) and APC anti-human CD25 (BioLegend, 356110) antibodies. To assess the expression of costimulatory activation markers, a separate aliquot of cells was stained with FITC anti-human CD80 (BioLegend, 375406) and Brilliant Violet 421™ anti-human CD86 (BioLegend, 305426) antibodies. The following isotype-matched controls were employed to account for non-specific binding: Brilliant Violet 421™ Mouse IgG2a, κ Isotype Ctrl Antibody (BioLegend, 400260), APC Mouse IgG1, κ Isotype Ctrl (FC) Antibody (BioLegend, 400122), FITC Rat IgG2a, κ Isotype Ctrl Antibody (BioLegend, 400506) and Brilliant Violet 421™ Mouse IgG2b, κ Isotype Ctrl Antibody (BioLegend, 400342). Data acquisition was performed on a flow cytometer (Agilent, 2010064AA). During analysis, single cells were gated using forward scatter area versus forward scatter height to exclude doublets. Following singlet gating, fluorescence compensation was applied to correct for spectral overlap. Targeted immune populations were identified based on the expression of surface markers, and all data were processed using the instrument's software.

- - 1. ***Flow Cytometric Analysis of Intracellular NF-κB and STAT3 Phosphorylation***

For the analysis of intracellular signaling pathways, OKT3 (BioXCell, BE0001-2) was diluted to 1 μg/mL in PBS and coated onto 24-well plates (1 mL/well) by incubation at 37°C for 1 h. Plates were washed twice with PBS. Human PBMCs (2 × 10⁶ cells/well) were seeded in RPMI 1640 (Gibco, 11875-093) supplemented with 10% (v/v) FBS and 1× antibiotic-antimycotic. The cells were concurrently stimulated with OKT3 and R848 (final concentration, 250 ng/mL) and treated with the bispecific antibody IDC007 (produced in-house) or control antibodies at a final concentration of 500 nM, achieving a total volume of 1 mL/well. Following 6 hours incubation at 37°C, 5% CO₂, cells were collected for intracellular phospho-protein analysis. The collected cells were fixed using BD Cytofix/Cytoperm™ Fixation and Permeabilization Solution (BD Biosciences) according to the manufacturer’s instructions. Following fixation, cells were permeabilized with BD Phosflow™ Perm Buffer III (BD Biosciences) on ice for 30 min. After permeabilization, the cells were stained with PE anti-NF-κB p65 Phospho (Ser529) antibody (BioLegend, 614154) or PE anti-STAT3 Phospho (Tyr705) antibody (BioLegend, 651004). All washing steps following fixation and permeabilization were performed using BD Perm/Wash™ Buffer (BD Biosciences). The following isotype-matched control was employed to account for non-specific binding: PE Mouse IgG1, κ Isotype Ctrl (ICFC) Antibody (BioLegend, 400140). Data acquisition was performed on a flow cytometer (Agilent, 2010064AA). During analysis, single cells were gated using forward scatter area versus forward scatter height to exclude doublets. The intracellular phosphorylation levels of NF-κB and STAT3 were quantified based on the mean fluorescence intensity of the corresponding phospho-specific antibodies, and all data were processed using the instrument's software.

- - 1. ***Transcription-factor activity analysis***

The public scRNA-seq dataset [GSE198868](https://www.ncbi.nlm.nih.gov/geo/query/acc.cgi?acc=GSE198868) was re-analyzed to assess NF-κB and STAT3 activation in an anti-HER2/CD3 T-cell-dependent bispecific antibody (TDB)-induced inflammatory model. Liver cells from vehicle- and TDB-treated mice bearing MMTV-HER2 Fo5 (Founder5) mammary tumors were analyzed using the original quality-filtered count matrix and cell annotation. For each mouse and major population, raw counts were aggregated into pseudobulk profiles and normalized as log1p-transformed counts per million. RELA/NF-κB and STAT3 activities were inferred using the Python package decoupler with CollecTRI mouse regulons. Vehicle and TDB groups were compared using Welch’s two-sided t-test, followed by Benjamini–Hochberg FDR correction across all cell population–transcription factor tests. FDR q < 0.05 was considered significant.

1. Supplementary figures


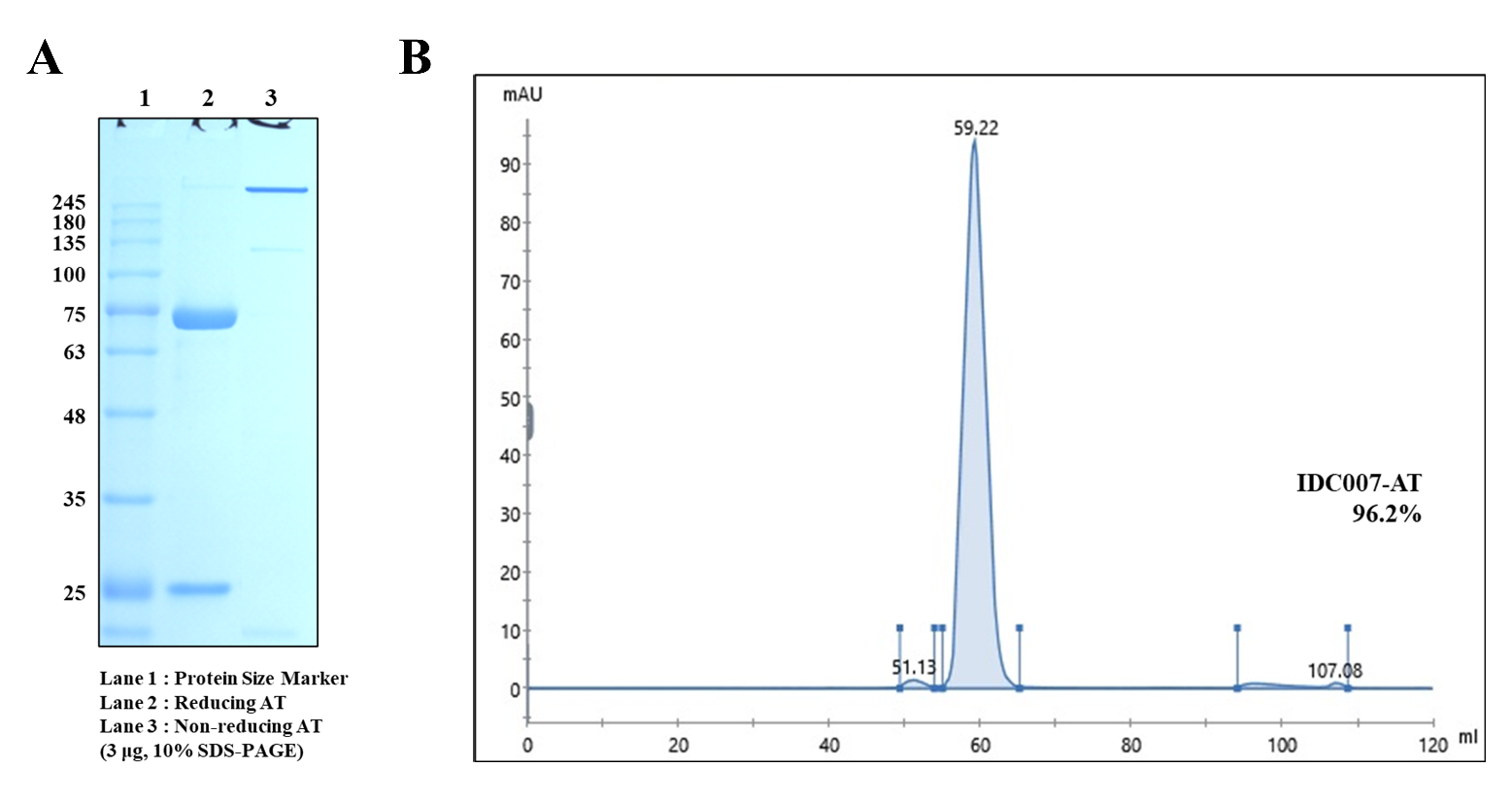


**Figure S1. Biochemical characterization and quality control of the purified IDC007 bispecific antibody. (A)** SDS-PAGE analysis of the purified IDC007 antibody. The analysis was performed under non-reducing and reducing conditions. A single intact band at approximately 198.8 kDa is observed under non-reducing conditions, representing the fully assembled bispecific antibody. Under reducing conditions, two distinct bands corresponding to the heavy chain-scFv fusion (76 kDa) and the light chain (23.4 kDa) are detected. **(B)** SE-FPLC profile of IDC007. The chromatogram demonstrates that the monomeric purity of the purified antibody is >95.0%.


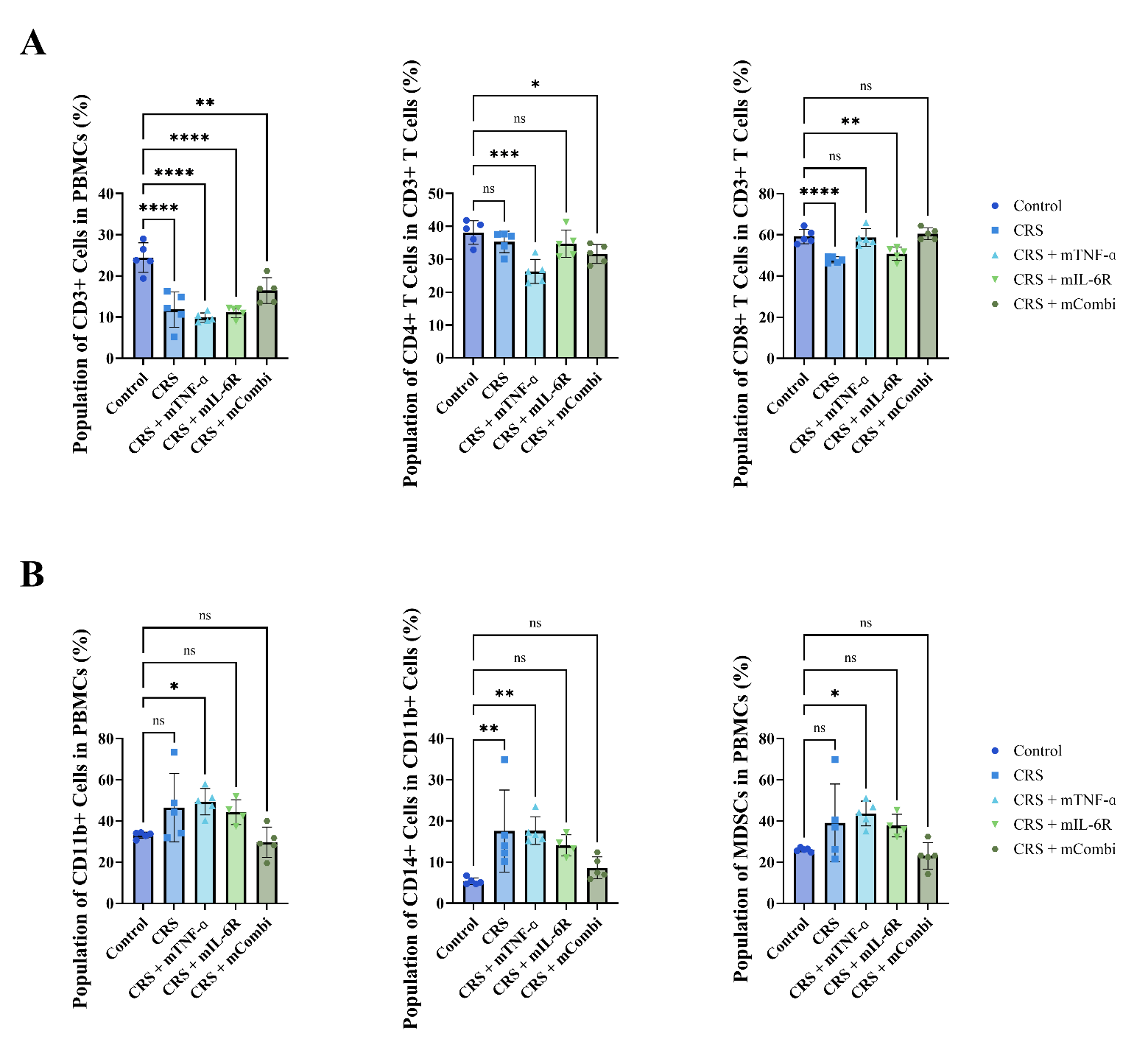


**Figure S2. Profiling of peripheral immune cell populations following CRS induction and treatment.** Flow cytometric analysis of immune cell populations in PBMCs. The proportions of **(A)** T-cell subsets (CD3^+^, CD3^+^ CD4^+^, and CD3^+^ CD8^+^ T cells) and **(B)** myeloid lineages (CD11b^+^, CD11b^+^ CD14^+^, and MDSCs) were quantified. Mouse surrogate antibodies (mTNF-α, mIL-6R, and their combination, mCombi) were used for the immunocompetent *in vivo* model. Data in **(A and B)** are presented as mean ± SD (n = 5 mice per group). Statistical significance was determined using one-way ANOVA followed by Dunnett’s multiple comparisons test. Notably, immune cell populations **(A and B)** were compared against the Control group to evaluate the restoration of immune homeostasis. * p < 0.05, ** p < 0.01, *** p < 0.001, **** p < 0.0001; ns, not significant.


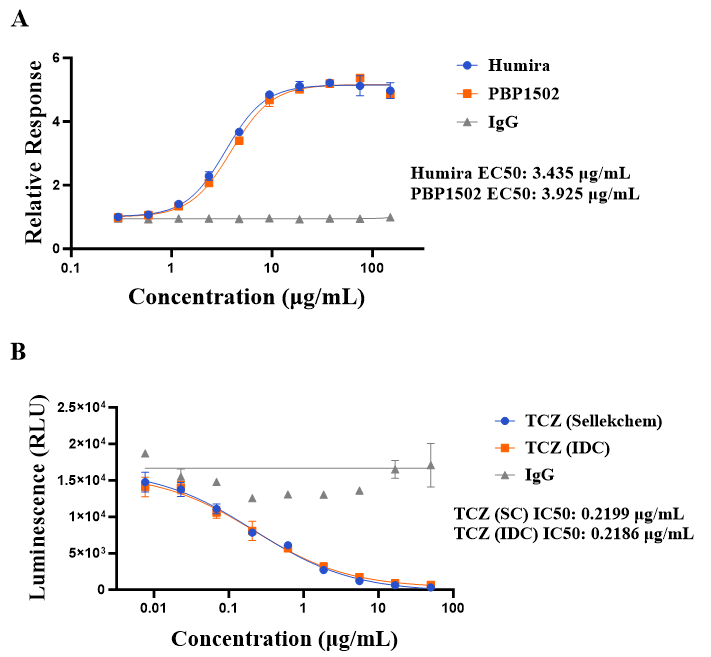


**Figure S3. Neutralizing potency of commercial reference and in-house parental antibodies.** **(A)** Cell-based functional assay demonstrating that the commercial reference adalimumab (Humira) and the in-house biosimilar (PBP1502) exhibit comparable potency in neutralizing TNF-α-induced cytotoxicity in Actinomycin D-sensitized WEHI-13VAR cells, with closely matched EC₅₀ values of 3.435 µg/mL and 3.925 µg/mL, respectively. A human IgG isotype was included as a baseline negative control, showing no neutralizing activity against TNF-α. **(B)** IL-6 bioassay demonstrating that the commercial tocilizumab (TCZ; Selleckchem) and the in-house produced tocilizumab (TCZ; IDC) exhibit equivalent potency in neutralizing IL-6-induced signaling, with closely matched IC₅₀ values of 0.2199 µg/mL and 0.2186 µg/mL, respectively. The IgG negative control exhibited no reduction in luminescence. Data in (A and B) are presented as the mean ± SD of 3 independent biological replicates (n = 3).


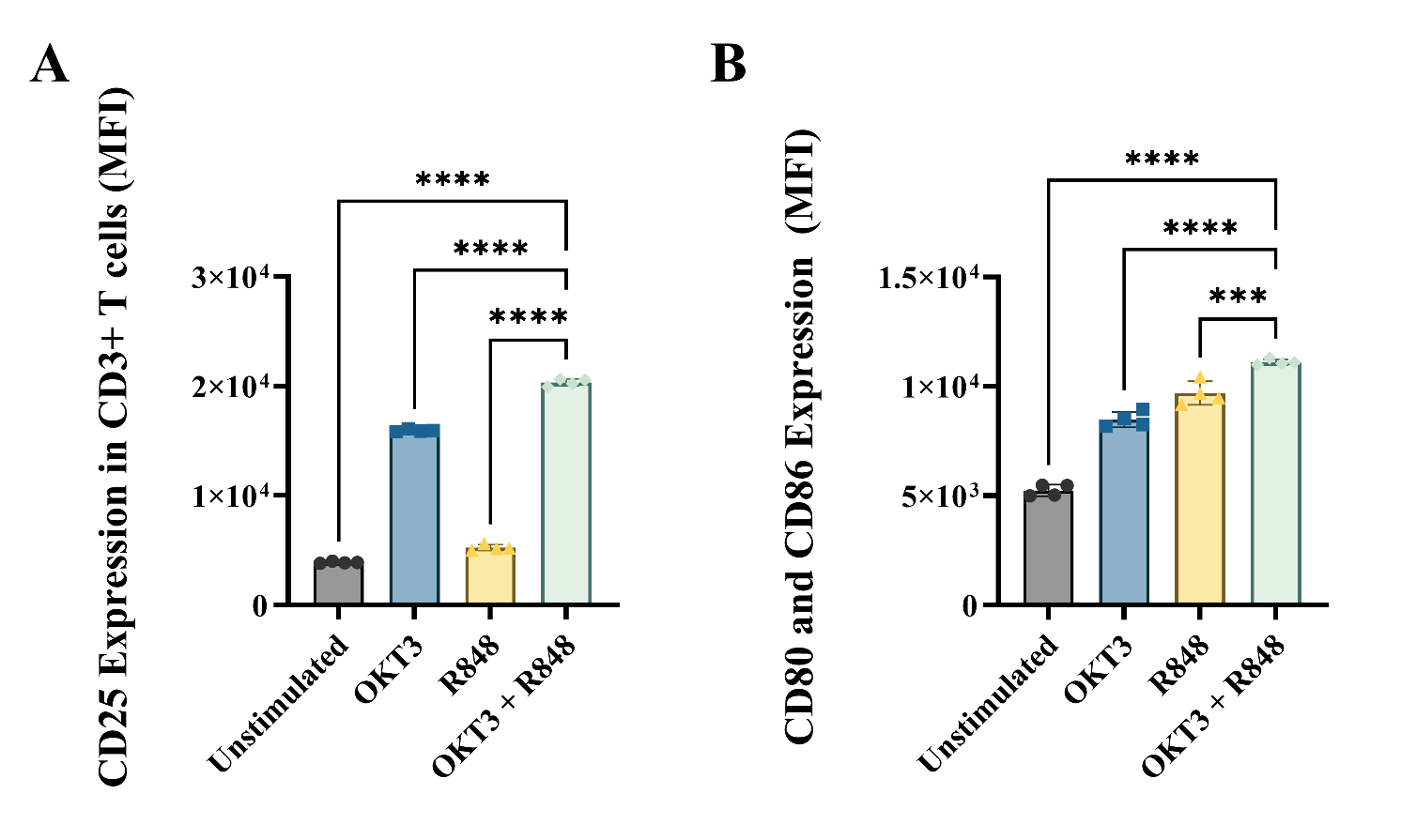


**Figure S4. Combined OKT3 and R848 stimulation synergistically activates hPBMCs.**

Human PBMCs were stimulated with OKT3 alone, R848 alone, or the combination of OKT3 and R848, and immune cell activation was analyzed by flow cytometry. **(A)** T-cell activation was assessed by evaluating the mean fluorescence intensity (MFI) of CD25 expression within the CD3^+^ population. **(B)** Expression of the co-stimulatory activation markers CD80 and CD86 was evaluated following stimulation. Combined stimulation with OKT3 and R848 induced significantly greater T-cell activation and CD80/CD86 expression than either stimulus alone, indicating enhanced immune cell activation under *in vitro* CRS-like conditions (n = 3). Data are presented as the mean ± SD of 3 independent biological replicates. Statistical significance was determined by one-way ANOVA followed by Tukey's multiple comparisons test. *** p < 0.001, **** p <0.0001.


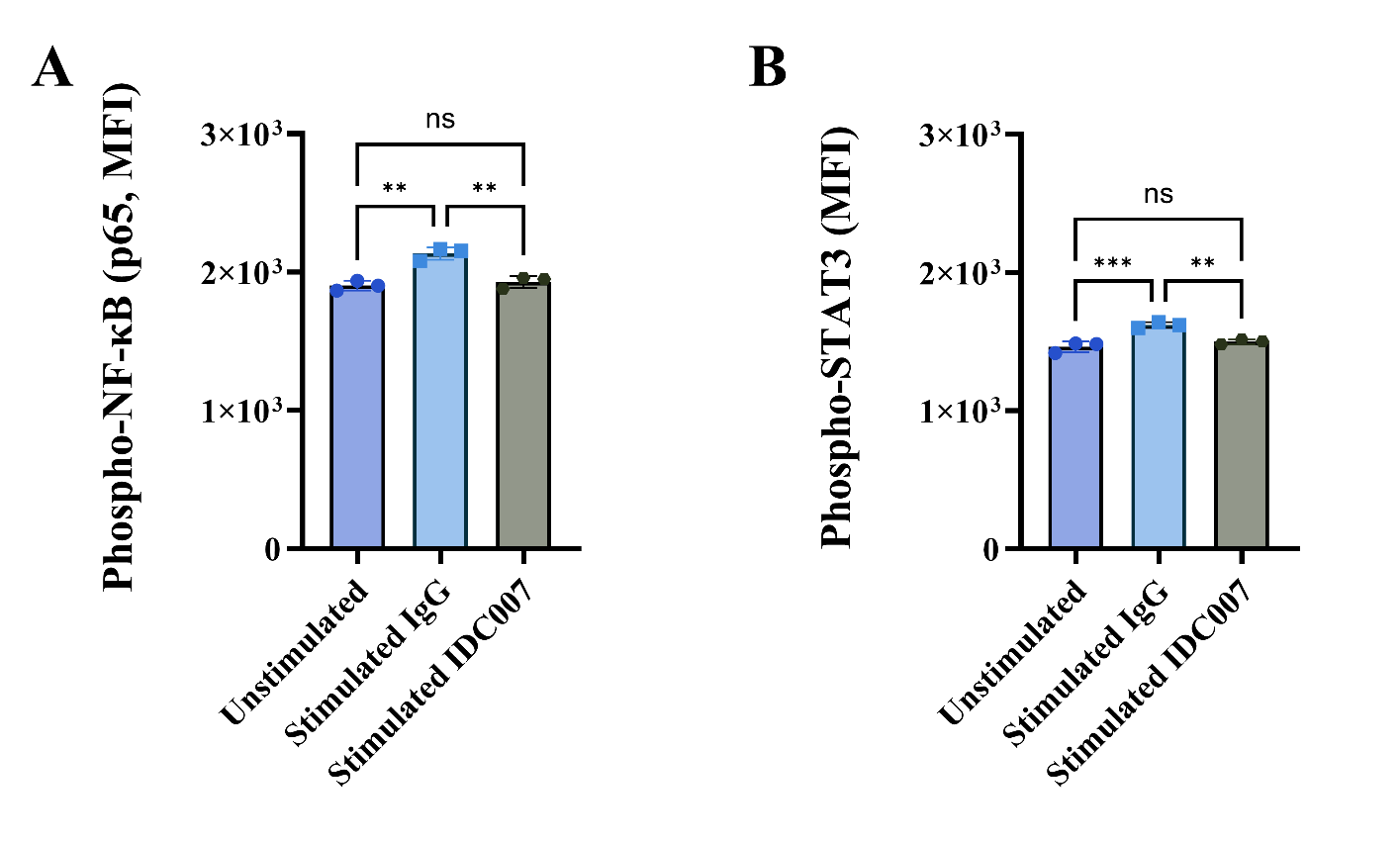


**Figure S5. IDC007 suppresses NF-κB and STAT3 signaling under simulated CRS conditions.**

Human PBMCs were either left unstimulated or stimulated with OKT3 and R848. Stimulated cells were concurrently treated with control IgG or IDC007. After 6 h, the intracellular levels of phosphorylated NF-κB **(A)** and STAT3 **(B)** were analyzed by flow cytometry. OKT3/R848 stimulation increased phospho-NF-κB and phospho-STAT3 levels. Treatment with IDC007 effectively reduced the phosphorylation of both transcription factors compared to the IgG group. Data are presented as mean ± SD of four independent biological replicates (n = 4). Statistical significance was determined by one-way ANOVA followed by Tukey's multiple comparisons test. ** p < 0.01, *** p < 0.001; ns, not significant.


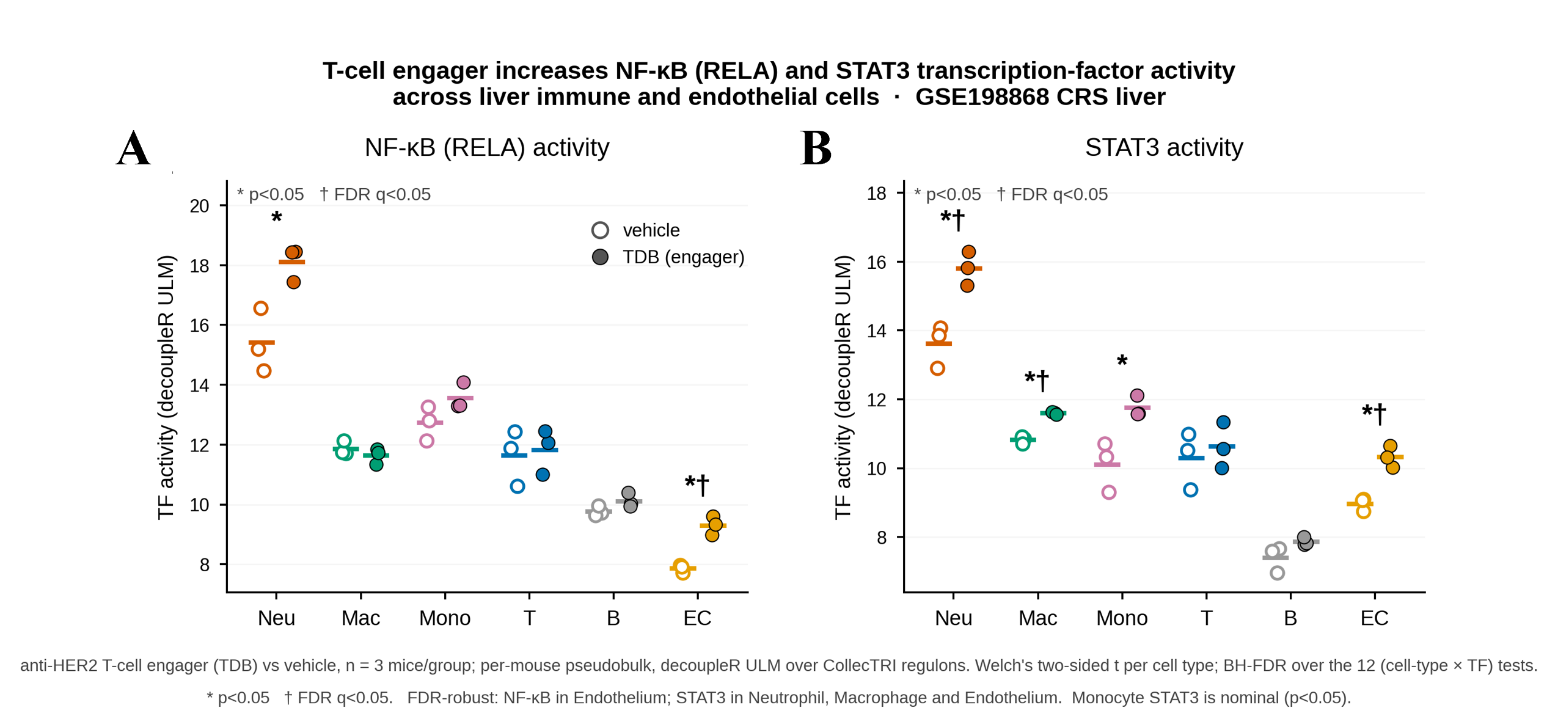


**Figure S6. T-cell engager treatment increases RELA/NF-κB and STAT3 activity in liver immune and endothelial cells.** Per-mouse pseudobulk transcription-factor activities were inferred from GSE198868 liver single-cell RNA sequencing (scRNA-seq) data using decoupler with CollecTRI regulons. **(A)** RELA/NF-κB and **(B)** STAT3 activities are shown for vehicle- and anti-HER2/CD3 T-cell-dependent bispecific antibody (TDB)-treated mice across six major cell populations (e.g., Mac, macrophages; Mono, monocytes; EC, endothelial cells). Open circles, vehicle; filled circles, TDB; horizontal bars, group means. *p < 0.05; †FDR (false discovery rate) q < 0.05.
